# Cluster analysis of cutting technique—a valuable approach for assessing anterior cruciate ligament injury risk?

**DOI:** 10.3389/fspor.2025.1463272

**Published:** 2025-02-10

**Authors:** Lasse Mausehund, Anri Patron, Sami Äyrämö, Tron Krosshaug

**Affiliations:** ^1^ Oslo Sports Trauma Research Center, Department of Sports Medicine, Norwegian School of Sport Sciences, Oslo, Norway; ^2^ Department of Computer Science, University of Helsinki, Helsinki, Finland; ^3^ Faculty of Information Technology, University of Jyväskylä, Jyväskylä, Finland; ^4^ Hospital Nova of Central Finland, Wellbeing Services County of Central Finland, Jyväskylä, Finland

**Keywords:** ACL, biomechanics, kinematics, kinetics, football, handball, K-means, return to sport

## Abstract

**Background:**

Despite extensive efforts to pinpoint singular biomechanical risk factors for anterior cruciate ligament (ACL) injuries, research findings are still inconclusive. By combining multiple biomechanical variables, cluster analyses could help us identify safe and risky cutting technique strategies.

**Purpose:**

To identify common movement strategies during cutting maneuvers and to assess their association with ACL injury risk.

**Methods:**

A total of 754 female elite handball and football players, including 59 with a history of ACL injury, performed a sport-specific cutting task while 3D biomechanics were recorded. Over an 8-year follow-up period, 43 of these players sustained a primary ACL injury and 13 players a secondary ACL injury. Cutting technique was described using 36 discrete kinematic variables. To identify different cutting techniques, we employed a K-means clustering algorithm on data subsets involving different numbers of kinematic variables (36, 13 and 5 variables) and different sports (handball, football, and both combined). To assess the impact of the identified cutting technique clusters on ACL injury risk, we compared the proportion of injured players between these clusters using the Fisher-Freeman-Halton Exact test and adjusted rand indices (ARI).

**Results:**

We identified two distinguishable cutting technique clusters in the subset involving both sports and five kinematics variables (average silhouette score, ASS = 0.35). However, these clusters were formed based on sport- or task-related differences (Fisher's *p* < 0.001, ARI = 0.83) rather than injury-related differences (Fisher's *p* = 0.417, ARI = 0.00). We also found two cutting technique clusters in the handball (ASS = 0.23) and football (ASS = 0.30) subsets with five kinematic variables. However, none of these clusters appeared to be associated with ACL injury risk (Fisher's *p* > 0.05, ARI = 0.00).

**Conclusion:**

No safe or risky cutting technique strategies could be discerned among female elite handball and football players. Cluster analysis of cutting technique, using a K-means algorithm, did not prove to be a valuable approach for assessing ACL injury risk in this dataset.

## 1 Introduction

Anterior cruciate ligament (ACL) injuries are a major concern in team ball sports, especially for female athletes, who face an incidence three times higher than their male counterparts (1). These injuries carry severe consequences not only for the injured athlete and their team but also for society at large. One of the most severe consequences of a primary ACL injury is the alarmingly high risk of suffering yet another ACL injury. As many as 1 out of every 5 athletes who return to play after an ACL injury will end up with a new ACL injury in the same or contralateral knee (2), with short- and long-term implications overshadowing those of a primary ACL injury (3–6). It is therefore critical to prevent such injuries and to make return to sport safer.

A first logical step in preventing ACL injuries is to investigate their underlying risk factors (7). We know that the risk of an ACL injury is multifactorial in nature (8–11) and we know that movement biomechanics might be one important piece of the puzzle (12). Since ACL injuries in handball (13, 14) and football (15, 16) most commonly occur during cutting maneuvers, identifying biomechanical risk factors in such movements is critical and may bring us one step closer to successful injury prevention. Even though a multitude of studies have assessed biomechanical risk factors for primary and secondary ACL injury in female athletes during various tasks, the research findings are still inconclusive. For example, some researchers have identified knee abduction angles and moments as risk factors for ACL injury (17), whereas others have not (18, 19). Some have identified stiff landings, meaning less knee flexion in combination with a greater knee flexion moment, as a risk factor (19, 20), whereas others have not (18). So far, not one single biomechanical variable has been consistently linked to ACL injury risk, including knee abduction angles and moments which are commonly accepted as unfavorable movement biomechanics (21). Therefore, other approaches might be necessary to give us new insights into biomechanical risk factors for ACL injury.

In conventional approaches, we commonly compare single biomechanical variables between groups with and without an injury or we assess associations between specific variables and future injury. However, sport-specific movements are complex, and it is likely that a combination of multiple kinematic and kinetic characteristics might impact ACL loading and injury risk rather than single variables. In contrast to conventional approaches, in cluster analysis approaches, we try to find patterns or groupings (i.e., clusters) in the whole group, independent of the injury status, and where the groupings are based on a combination of multiple biomechanical variables. Thus, cluster analysis methodology allows us to identify and characterize typical movement patterns. Potentially, there are several different, commonly used movement patterns during cutting maneuvers, which we will refer to in this study as cutting technique clusters. For example, one cutting technique cluster might involve more torso lateral flexion in combination with more knee valgus and less ankle plantar flexion as compared to another cluster. After identifying cutting technique clusters, we can assess if injured players are more represented in one of the clusters, indicating that this specific cutting technique strategy may lead to a higher risk of ACL injury. Thereby, cluster analysis can help us identify safe and risky cutting technique strategies, where the risky strategies can be targeted in interventions. Using cluster analysis, two previous studies have successfully identified distinct movement strategies during jumping tasks, some of which were found to be associated with increased knee abduction moments (22), a proxy for ACL injury risk, or increased musculoskeletal injury risk (23). To the best of our knowledge, there are no studies using cluster analysis techniques to identify movement strategies during cutting maneuvers which are associated with ACL injury risk.

To investigate potential biomechanical risk factors for ACL injury in female elite football and handball players this study had two purposes. The first purpose was to identify the most common cutting technique clusters based on pre-defined 3D kinematic variables from baseline testing. The second purpose was to determine if the identified cutting technique clusters were associated with ACL injury risk in players, both with and without previous ACL injury, during the follow-up period. In addition, we aimed to assess differences in knee abduction moments across the identified clusters.

## 2 Materials and methods

### 2.1 Study design and participants

In this investigation we applied cluster analysis methodology (unsupervised classification) to determine a set of different cutting techniques with biomechanically different characteristics and we assessed their influence on ACL injury risk in players with and without previous injury. This study is part of a comprehensive prospective cohort study focused on identifying risk factors for non-contact ACL injuries among female elite handball and football players (18, 24–26).

**Figure 1:**
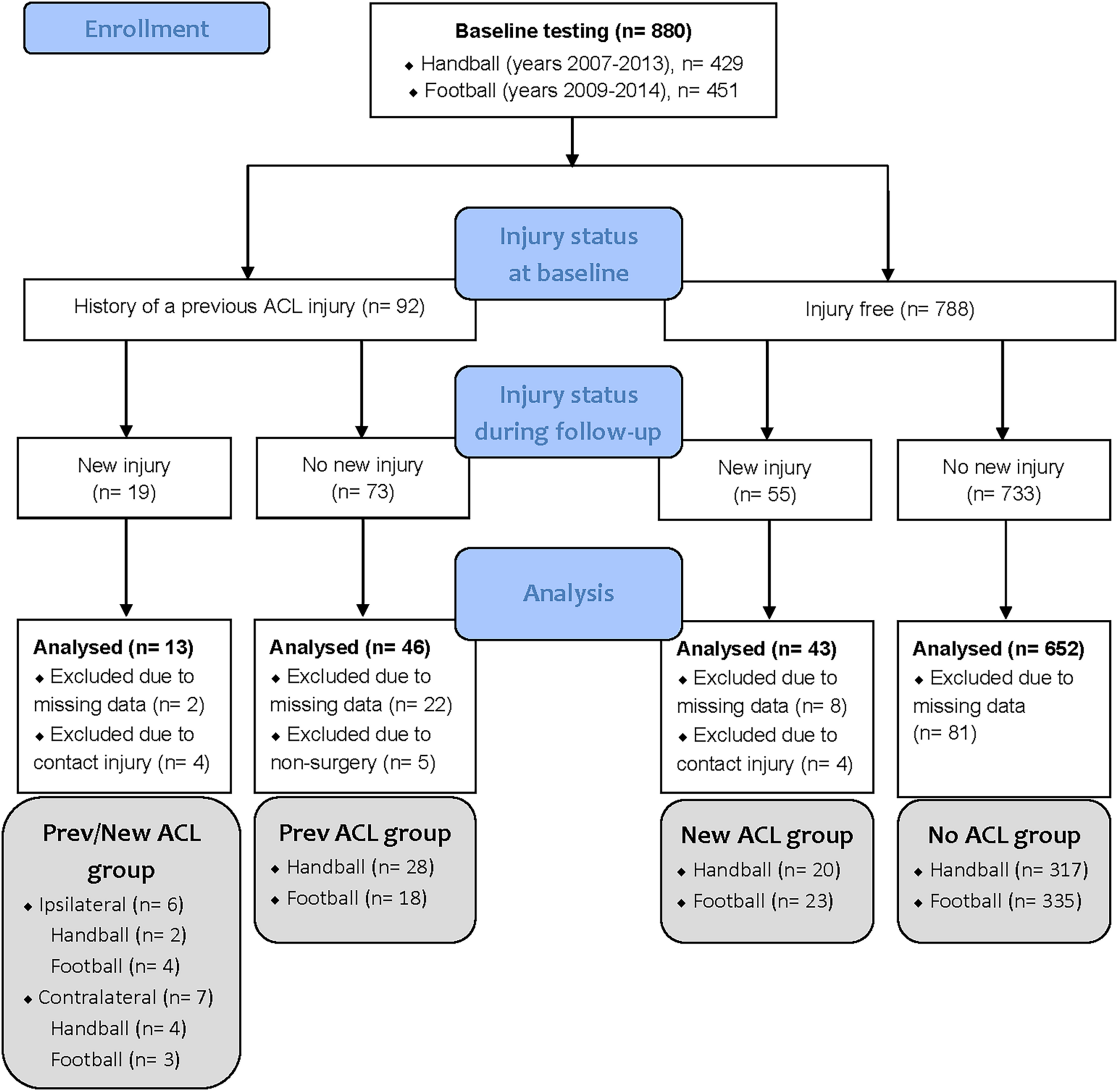
Flow diagram of the tested players, including their injury status at baseline and follow-up, as well as the number of analyzed players in each group. ACL, anterior cruciate ligament.

All data were gathered over an 8-year period, beginning in 2007, when all clubs in Norway's elite female handball league were invited to participate in an extensive preseason baseline testing. To be included, players were required to have a first-team contract and were expected to play in the Premier League during the upcoming season. From 2008 to 2013, players from new Premier League teams as well as new players from existing teams were subjected to the same preseason baseline test. Female Premier League football players were also included in the study from 2009 to 2014, using the same inclusion criteria. A total of 880 athletes were tested, including 451 football and 429 handball players. During the follow-up period, 15 participants suffered secondary non-contact ACL injuries, while 51 suffered primary non-contact ACL injuries. In the current study, we excluded players with direct contact–related new ACL injuries (*n* = 8) as well as players with a previous ACL injury who had not undergone ACL reconstruction surgery (*n* = 5). Also, thirteen percent of the players (*n* = 113) had to be excluded due to missing kinematic data in the cutting task, which was caused by technical problems, illness or injury. Thus, the final sample consisted of 754 players (age, 20.7 ± 3.9 years; body mass, 66.2 ± 7.9 kg; height, 169.5 ± 6.3 cm), who were subdivided into the following four groups: Players with a previous ACL injury who sustained a new, secondary ACL injury during follow-up (Prev/New ACL group), players with a previous ACL injury only (Prev ACL group) and players without a previous ACL injury who did (New ACL group) or did not (No ACL group) sustain a new, primary ACL injury during follow up (Figure 1). The previously ACL injured players (*n* = 59) were tested, on average, 3.6 ± 2.4 years after their injury.

The study was approved by the Regional Committee for Medical Research Ethics, the Regional Health Authority of South-Eastern Norway, and the Norwegian Social Science Data Services. Before inclusion, all participants signed a written informed consent form, which included parental consent for players under the age of 18. The study adhered to the most recent iteration of the Helsinki Declaration.

### 2.2 Data collection and test procedures

During the preseason, all players attended one day with extensive baseline testing, including a vertical drop jump task, a cutting task and a variety of neuromuscular, mobility, clinical and anthropometrical assessments. The foundation for this study was a biomechanical analysis of sport-specific cutting tasks using three-dimensional motion capture. The details of the marker placement and cutting test procedure have been described previously (27, 28). For the handball-specific cut, the player received a lateral pass from a teammate right before performing a match-like cutting maneuver to fake and pass a human static defender. For the football-specific cut, the player received a football pass forcing her to perform a sharp sidestep cutting maneuver. For each leg, at least five successful trials with maximum match-like effort were recorded, with the first three being chosen for analysis. Trials 4 and 5 were considered viable alternatives if one or more markers were hidden during parts of the cutting movement or if the force platform was partially missed.

Following baseline testing, all complete ACL ruptures were registered prospectively through May 2015, mostly through regular contact with the participating teams’ managers, coaches, or medical staff. In the event of an acute knee injury, we contacted the injured player directly to get medical information and a description of the injury situation. The mechanism of injury was self-classified as contact, indirect contact, or non-contact. Magnetic resonance imaging and/or arthroscopy were used to confirm all complete ACL ruptures.

### 2.3 Measurements and data processing

All measurements were collected synchronously using a 16-bit analog-to-digital conversion board (USB-2533; Measurement Computing Corporation, Norton, MA, USA), then integrated into Qualisys Track Manager (version 2.8; Qualisys AB, Gothenburg, Sweden) and processed in Matlab (version 2011; MathWorks Inc., Natick, MA, USA). Three-dimensional kinematic data were collected from 2007 to 2012 using an 8-camera motion capture system (ProReflex; Qualisys AB, Gothenburg, Sweden) sampling at 240 Hz. Since 2012, an updated 16-camera system with a sample frequency of 480 Hz was employed (Oqus 4; Qualisys AB). Ground reaction forces and center of pressure were measured with two force platforms (AMTI LG6-4-1, Watertown, MA, USA) at 960 Hz. Data processing, including filtering, interpolation, joint center position estimation as well as joint angle and moment calculations have been described previously (18, 28).

Sidestep cutting technique was described using 36 kinematic variables, including 3D hip, knee and ankle angles at initial contact as well as peak angles during the ground contact phase. At initial contact, 3D torso angles, cutting width and depth, foot to floor angles, cutting angle and approach speed were also used to describe cutting technique. Finally, the time at which the peak angles occurred as well as ground contact time were also included. Cutting width and depth were defined as the respective angles between a line from the center of mass to the center of pressure and a vertical line in a plane perpendicular to the direction of movement 20 ms after initial contact (29). Torso flexion, lateral flexion and rotation were defined as angles relative to the ground and direction of movement at initial contact. For each leg, the mean of the three cutting trials served as the foundation for all analyses. In order to give equal weights to all the input biomechanical variables in the cluster analyses, they were normalized by linear scaling into the closed interval [0, 1].

### 2.4 Cluster analyses

#### 2.4.1 Dataset subdivision

To determine if the biomechanical variables can be used to cluster safe and risky cutting techniques, the dataset was initially divided into different subsets (Tables 1, 2): First, four subsets were based on how many biomechanical variables were included in the model, whereof one subset involved all kinematic variables described above (All 36), one subset involved a reduced selection of 13 kinematic variables at initial contact (Core 13: Cutting width and depth, 3D torso angles relative to the ground and direction of movement, hip abduction and rotation angles, knee flexion and valgus angles, foot to floor angle and foot rotation angle relative to direction of movement, approach speed and cutting angle) and one subset narrowed the selection down to 5 kinematic variables at initial contact (Core 5: Cutting width and depth, 3D torso angles relative to the ground and direction of movement). The core selections were based on expert-opinion and previous research (17, 19, 29, 30), and they included variables which have been shown to be related to ACL injury risk, or at least to knee abduction moments, and variables which are more easily modifiable through training. We selected variables occurring at initial contact because ACL injuries typically occur approximately 40 ms after initial contact (31), making these variables highly relevant and in many cases more pertinent than, for example, peak values, which often occur later during the stance phase. We chose to focus on kinematic variables only, since these are more readily modifiable during interventions and do not necessitate force plates for measurement. Our aim was to make the identification of safe and potentially risky cutting technique strategies more practical in real-world application. The last subset involved dimensionality reduction using principal component analysis (PCA). PCA was applied as a computational approach to reduce the total number of variables (i.e., 36 dimensions), effectively transforming the original data into the lowest dimensional projection possible while still preserving a minimum of 80% of the total data variance. Twelve principal components (i.e., 12 dimensions) were required to achieve the necessary variance in each subset. PCA was used to simplify the data, reduce noise, improve cluster quality and performance, mitigate multicollinearity, and help the clustering algorithm to identify meaningful patterns more effectively.

**Table 1:** Cluster analysis methodology was applied to 24 unique data subsets which were based on three different categories. Each subset from the first category was combined with every possible combination of subsets from the other two categories (4 × 3 × 2 = 24 subsets in total). A detailed explanation can be found in the text.

4 subsets based on number of variables	3 subsets based on sport	2 subsets based on testing leg
All 36 kinematic variables (All 36)	Handball and football players combined	Ipsilateral leg subset: - Prev/New ACL group: leg with new ipsilateral re-injury - Prev ACL group: ipsilateral leg - New ACL group: leg with new injury - No ACL group: random leg
Core selection of 13 kinematic variables (Core 13)	Handball players only	Contralateral leg subset - Prev/New ACL group: leg with new contralateral injury - Prev ACL group: contralateral leg - New ACL group: leg with new injury - No ACL group: random leg
Core selection of 5 kinematic variables (Core 5)	Football players only	
12 principal components based on PCA (PCA)		

All 12 ipsilateral subsets are listed in Table 2. The corresponding 12 contralateral subsets can be found in the Supplementary Material 1; PCA, principal component analysis; Prev/New ACL group, players with a previous ACL injury who went on to sustain a new secondary ACL injury; Prev ACL group, players with a previous ACL injury only; new ACL group, players without a previous ACL injury who went on to sustain a new primary ACL injury; no ACL group, injury free players.

**Table 2:** Average silhouette scores for all subsets and for different number of clusters. Only the ipsilateral leg subsets are presented.

	2 clusters	3 clusters	4 clusters	5 clusters
All 36	**0**.**16**^a^	0.14	0.13	0.11
All 36 (PCA)	**0**.**19**^a^	0.15	0.14	0.13
Core 13	**0**.**22**^a^	0.18	0.16	0.14
Core 5	**0.35** ^a^ **^,^** ^b^	0.29^b^	0.25^b^	0.21
Handball all 36	**0**.**13**^a^	0.09	0.08	0.07
Handball all 36 (PCA)	**0**.**13**^a^	0.11	0.11	0.09
Handball core 13	**0**.**13**^a^	0.13	0.12	0.11
Handball core 5	**0.23** ^a^ **^,^** ^b^	0.20	0.17	0.16
Football all 36	**0**.**14**^a^	0.10	0.08	0.07
Football all 36 (PCA)	**0**.**15**^a^	0.12	0.10	0.09
Football core 13	**0**.**18**^a^	0.13	0.11	0.11
Football core 5	**0.30** ^a^ **^,^** ^b^	0.21	0.20	0.19

The first four subsets involve both handball and football players, as well as different selections of kinematic variables [all 36 variables, a narrowed selection of 13 and 5 variables, and a principal component analysis (PCA) reduction of all 36 variables resulting in 12 principal components]. The last eight subsets involve either handball or football players alone and the same selection of variables.

^a^ The highest average silhouette score of all cluster models within the same subset (bold used for visibility).

^b^ Subsets with an average silhouette score exceeding 0.25, indicating some evidence of cluster existence, and subsets scoring highest within their sport group (bold used for visibility).

Second, three subsets were defined by sport: one with both handball and football players, one with only handball players, and one with only football players.

Third, two subsets were based on the testing leg. Since players with an ACL injury history have an increased risk of a new injury in both the ipsilateral and contralateral knee (2), both legs were included in the analyses. However, since previous research indicates that risk factors for ipsilateral re-injury and contralateral injury might differ (10, 11, 32, 33), we conducted separate analyses for the ipsilateral leg and ipsilateral re-injury as well as the contralateral leg and contralateral injury. Therefore, in one subset, the Prev/New ACL group included only players who sustained a new ipsilateral re-injury during follow-up, and for the Prev ACL group, we chose the ipsilateral leg (ipsilateral leg subset). In the other subset, the Prev/New ACL group included only players who sustained a new contralateral injury, and for the Prev ACL group, we selected the contralateral leg (contralateral leg subset). In both subsets, we chose the leg which sustained the new injury during follow-up for the New ACL group, and a randomly selected leg for the No ACL group.

#### 2.4.2 Identification of cutting technique clusters

For clustering these data subsets, a partition-based K-means clustering algorithm was applied. In order to avoid convergence to local minima, we used K-means++ initialization, which attempts to spread out the initial cluster centers (34). Each data point was assigned to the nearest cluster center based on the Euclidean distance (35). The cluster centers were then updated by recomputing the mean of the data points assigned to each cluster (35). This operation was repeated until convergence, defined according to the Frobenius norm as cluster centers changing less than 1e-10 between consecutive iterations, or until a maximum count of 1,000 iterations was reached. The algorithm was set to run 10 different initializations, the model with the lowest measure of the squared sum between data points and nearest cluster center was chosen as the final model. We chose a k-means clustering algorithm due to its efficiency and easily interpretable results. Our intention was not to find any arbitrary clustering structure, but specifically to identify the “most common” cutting technique movement strategies. The k-means algorithm seemed to be a highly suitable choice for this purpose, as it inherently presumes clusters of comparable sizes and densities, increasing the likelihood of identifying “common” strategies. Given the exploratory nature of our study, we aimed to balance model complexity and interpretability, which k-means allowed us to achieve effectively. We did not explore additional clustering methods to avoid the risk of overfitting and the risk of identifying appealing yet spurious patterns.

To determine the number of clusters for the K-means algorithm, several models with different number of clusters [k = 2,…,5] were fitted, and the optimal number of clusters was selected by applying silhouette analyses, using the Euclidean distance as a similarity metric (36). The silhouette score ranges between [−1; 1], where close to 1 implies that the data point lies well within its cluster, a value of 0 implies that the data point lies somewhere in between to two neighboring clusters and a negative value implies that the data point is assigned to the wrong cluster (37). The average silhouette score of a cluster model reflects how well the cluster model is able to separate the data (37). Therefore, the number of clusters with the highest average silhouette score was selected for the K-means algorithm. Since the two cluster models achieved the highest average silhouette score for all subsets (Table 2), the K-means algorithm was seeded with two clusters for all the models. Further, the average silhouette score can be used as evidence of cluster existence. According to Larose and Larose (38), an average silhouette score of less than 0.25 is an insufficient indication of cluster existence, whereas a score of 0.5 or higher is good evidence of the reality of the clusters in the data, indicating that clusters are clearly distinguishable. A score between 0.25 and 0.5 indicates some evidence of the existence of the clusters in the data, but domain-specific expertise is important to support the reality of these clusters (38).

For each subset with at least some evidence of cluster existence (i.e., average silhouette score ≥0.25) and for each subset scoring highest within its sport category, the following data analyses were conducted to further assess the reality of the clusters as well as their importance with regards to ACL injury risk: The cutting technique of each identified cluster were presented by the cluster mean ± SD of each biomechanical variable included in the model. For further interpretation of the cluster model, *t*-tests were performed for all the input variables. Due to the descriptive nature of the cluster analysis, it is important to be aware that the aim of the *t*-tests was not hypothesis testing *per se*, but help to identify which input variables separate the two clusters the most. Hence, t-statistics and Cohen's d effect size were used to determine the ranking of the input variables. Normality was assumed based on the Central Limit Theorem. Levene's test was used to assess the equality of the group (i.e., cluster) variances. If one or more variables in a cluster model were found heterogeneous, Welch's *t*-tests were performed. If all variables were found homogeneous, Student's two sample *t*-tests were conducted. The same *t*-tests were conducted for all variables in a cluster model to ensure comparability of the t-statistics. The level of significance was set *a priori* at *p* ≤ 0.05.

#### 2.4.3 Cutting technique clusters and ACL injury risk

To determine if the identified cutting technique clusters were of relevance for ACL injury risk, we applied Fisher-Freeman-Halton Exact tests (with a significance limit of *p* ≤ 0.05) and calculated adjusted rand indices for the four injury groups depicted in Figure 1. The Fisher-Freeman-Halton Exact test assessed the statistical association between the clusters and the injury groups, while the adjusted rand index evaluated the distribution of the players across the clusters. When applicable, we also performed these analyses for the two sport groups (handball and football), to assess if the clusters were based on sport-related differences rather than on differences related to injury status. The adjusted rand index objectively measures the similarity between two different clusterings of the same data set (39, 40). It ranges between [−1,1], with scores close to 0 indicating an agreement that is no better than random, scores close to 1 indicating perfect agreement between the two clusterings and scores close to −1 indicating complete disagreement (39, 40). In this study, the adjusted rand index was used to evaluate if the clustering results agree with the true labels of the injury groups and the sport groups (if applicable) (41). Since only two clusters were identified in each subset, despite four injury groups being present, three new binary injury groups (BIN groups in Table 3) were derived from the original injury groups and tested against the clustering results to assess if two or more of the original injury groups were clustered together. Finally, we also performed *t*-tests to assess between-cluster differences in peak knee abduction moment, which is often considered a main biomechanical risk factor for ACL injury (42). Cluster analyses and all statistical tests were performed using Python (Python version 3.12.2; The Python Software Foundation), except for the Fisher-Freeman-Halton Exact test, which was conducted in IBM SPSS Statistics (version 24; IBM Corporation, Armonk, NY, USA).

**Table 3:** Adjusted rand indices comparing the clustering results with the true labels of four different injury groupings and one sport grouping.

	Injury groups	BIN No ACL group	BIN Prev ACL group	BIN New ACL group	Sport groups
Core 5	0.00	0.00	0.00	0.00	0.83
Handball core 5	0.00	0.00	0.00	0.00	n.a
Football core 5	0.00	0.00	0.01	0.00	n.a

Injury groups, the four injury groups depicted in Figure 1; BIN No ACL group, no ACL group vs. all other groups; BIN prev ACL group, prev/new ACL group and prev ACL group vs. all other groups; BIN new ACL group, prev/new ACL group and new ACL group vs. all other groups; sport groups, handball vs. football players.

## 3 Results

The results of the cluster analyses for the ipsilateral and contralateral leg subsets were close to identical. For clarity, only the results of the ipsilateral leg subsets are presented. The results of the contralateral leg subsets are appended (Supplementary Material 1).

The average silhouette scores for all data subsets and cluster models were relatively low (<0.50), implying that no clearly distinguishable cutting technique clusters could be identified (Table 2). Only the Core 5 subset and the Football Core 5 subset demonstrated some evidence of cluster existence, as indicated by an average silhouette score of at least 0.25. The Core 5 subset, the Football Core 5 subset and the Handball Core 5 subset achieved the highest scores within their respective sport groups. Hence, further analyses were conducted for those three subsets only.

**Figure 2:**
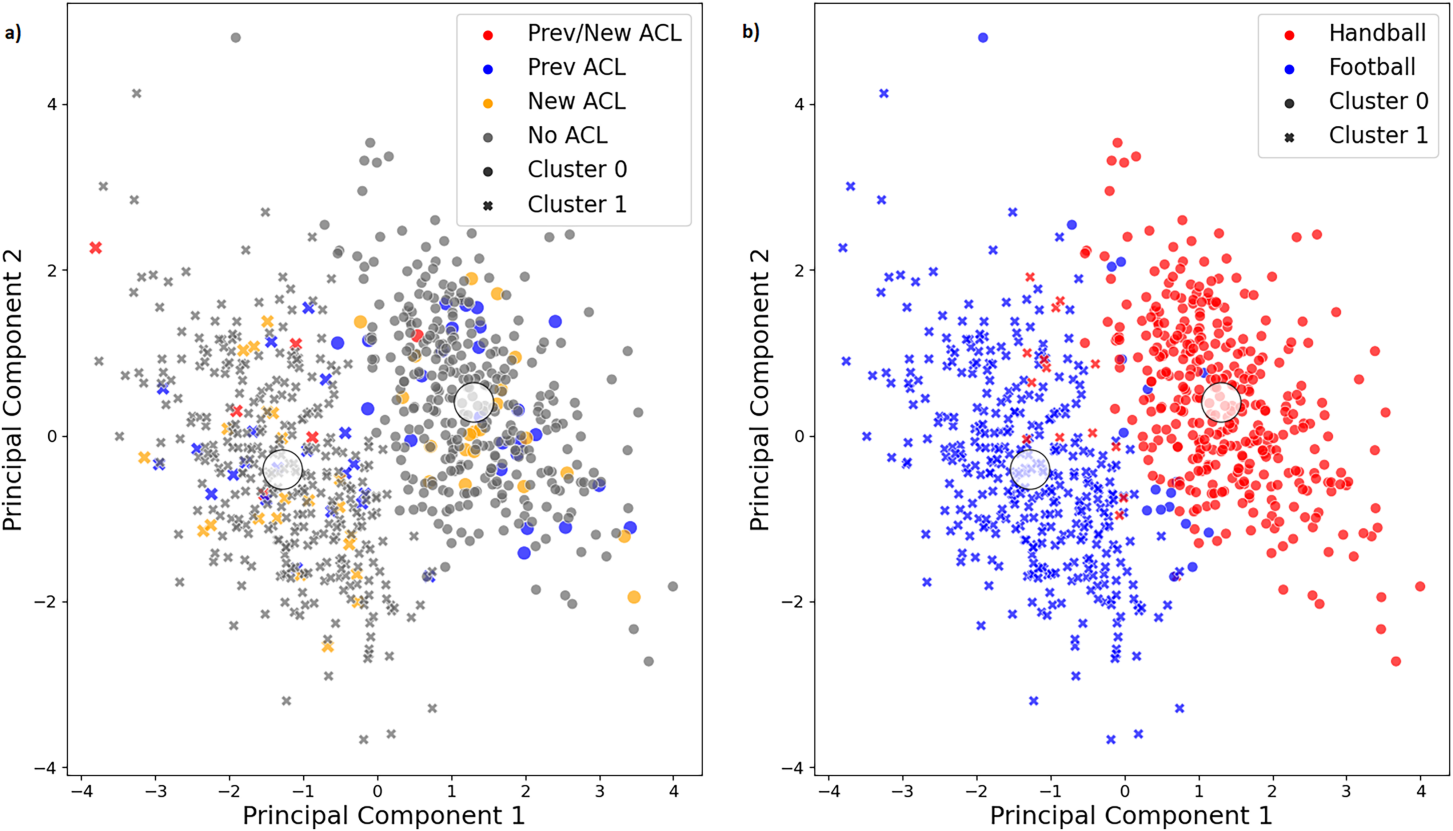
Visualization of the two clusters (cluster 0 and cluster 1) as well as the true labels of **(a)** the injury groups and **(b)** the sport groups for the core 5 subset. The first two principal components of the principal component analysis, which explain the highest amount of variance in the original data, are plotted against each other. Prev/New ACL group, players with a previous ACL injury who went on to sustain a new secondary ACL injury; Prev ACL group, players with a previous ACL injury only; new ACL group, players without a previous ACL injury who went on to sustain a new primary ACL injury; no ACL group, injury free players.

Descriptive and inferential statistics for the Core 5 subset containing both sports are presented in Table 4. The three input variables which separated the two identified cutting technique clusters the most were torso flexion followed by cutting width and torso lateral flexion (Table 4). The clusters were not significantly associated with the ACL injury groups (Fisher's *p* = 0.417; Table 5). The players appeared to be randomly distributed across the two clusters (Figure 2), as indicated by adjusted rand indices equal to zero for the four injury groups and their binary derivates (Table 3). Instead, the cutting technique clusters were associated with the sport groups (Fisher's *p* < 0.001). As can be seen in Table 6; Figure 2, the majority of the players in Cluster 0 were handball players, whereas football players were mainly assigned to Cluster 1. This is supported by a high adjusted rand index of 0.83, indicating high agreement between the clustering results and the true labels of the sport groups (Table 3). Interestingly, the peak knee abduction moments were significantly higher (*p* < 0.001; mean difference, 0.20 Nm/kg; Cohen's d, 0.37) in Cluster 1 (1.74 ± 0.57 Nm/kg) as compared to Cluster 0 (1.54 ± 0.51 Nm/kg).

**Table 4:** Cluster descriptive and inferential statistics for the core 5 subset.

	Cluster 0 (*n* = 370)	Cluster 1 (*n* = 377)	MD	*p*-value	Welch t-statistic	Cohen's d
Cutting width (°)	20.4 ± 3.9	28.6 ± 4.2	8.1	<0.001*	27.43	2.01
Cutting depth (°)	25.9 ± 4.7	32.4 ± 5.0	6.5	<0.001*	18.38	1.34
Torso flexion (°)	−9.3 ± 9.3	11.8 ± 9.9	21.1	<0.001*	30.08	2.20
Torso lateral flexion (°)	1.4 ± 7.9	13.5 ± 8.4	12.2	<0.001*	20.43	1.49
Torso rotation (°)	3.8 ± 13.6	12.0 ± 10.6	8.3	<0.001*	9.26	0.68

Values are means ± SD. MD, mean difference.

* Significant mean difference (*p* ≤ 0.05). Torso flexion: positive values indicate torso forward flexion; torso lateral flexion: positive values indicate torso lateral flexion in the intended cutting direction; torso rotation: positive values indicate torso rotation in the intended cutting direction (bold used for visibility).

**Table 5:** The distribution of players between the two clusters in each injury group for the core 5 subset.

	Prev/New ACL group	Prev ACL group	New ACL group	No ACL group
Cluster 0 (*n* = 370)	1 (0.3%)	25 (6.8%)	21 (5.7%)	323 (87.3%)
Cluster 1 (*n* = 377)	5 (1.3%)	21 (5.6%)	22 (5.8%)	329 (87.3%)

Values are number of players (percentage of *n*); the Fisher-Freeman-Halton exact test yielded a *p*-value of 0.417; Prev/New ACL group, players with a previous ACL injury who went on to sustain a new secondary ACL injury; Prev ACL group, players with a previous ACL injury only; new ACL group, players without a previous ACL injury who went on to sustain a new primary ACL injury; no ACL group, injury free players.

**Table 6:** The distribution of players between the two clusters in each sport group for the core 5 subset.

	Football	Handball
Cluster 0 (*n* = 370)	18 (4.9%)	352 (95.1%)
Cluster 1 (*n* = 377)	362 (96.0%)	15 (4.0%)

Values are number of players (percentage of *n*); The Fisher-Freeman-Halton exact test yielded a *p*-value of <0.001.

**Figure 3:**
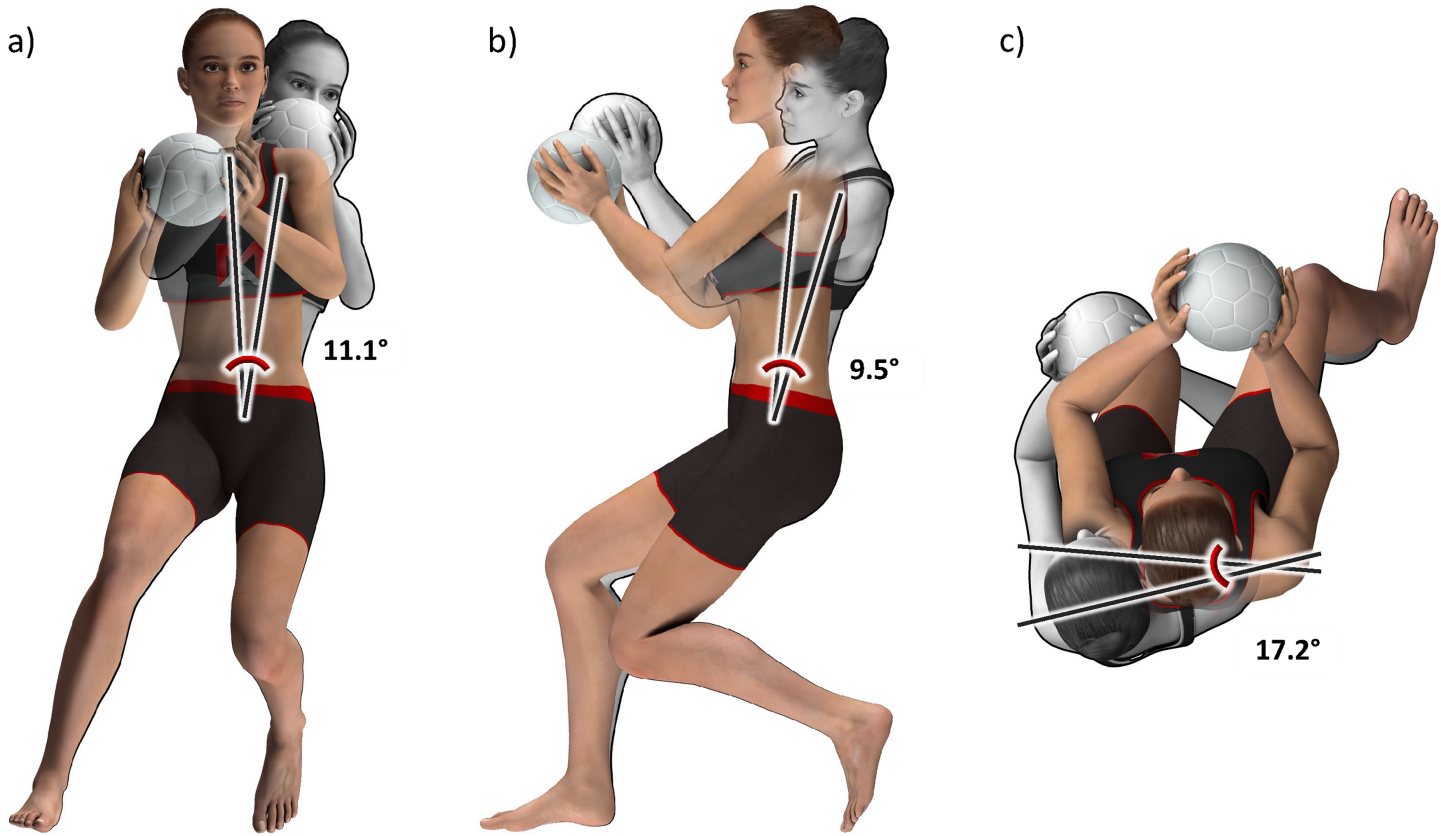
Animated figures for the handball core 5 subset, illustrating the mean differences in cutting technique between cluster 0 (grey) and cluster 1 (color) in **(a)** the frontal plane, **(b)** the sagittal plane, and **(c)** the horizontal plane. The players nearest to the respective cluster centers, established by the Euclidean norm, were chosen as the basis. Created by Muscle Animations.

Descriptive and inferential statistics for the Handball Core 5 subset are presented in Table 7; Figure 3. The three input variables which separated the two clusters the most were torso lateral flexion followed by torso rotation and torso flexion (Table 7). Again, these cutting technique clusters were found to be irrelevant to ACL injury risk (Fisher's *p* = 0. 836; Table 8). The player distribution seemed to be random across the two clusters, as confirmed by adjusted rand indices close to zero for all injury groupings (Table 3). Also, the peak knee abduction moments did not differ significantly between clusters (Cluster 0, 1.55 ± 0.51 Nm/kg; Cluster 1, 1.55 ± 0.52 Nm/kg; *p* = 0.917; mean difference, 0.01 Nm/kg; Cohen's d, 0.01).

**Table 7:** Cluster descriptive and inferential statistics for the handball core 5 subset.

	Cluster 0 (*n* = 193)	Cluster 1 (*n* = 174)	MD	*p*-value	Welch t-statistic	Cohen's d
Cutting width (°)	19.8 ± 4.2	21.1 ± 3.2	1.3	**0**.001*	3.34	0.35
Cutting depth (°)	27.1 ± 4.4	24.6 ± 4.6	2.4	**<0**.001*	5.16	0.54
Torso flexion (°)	−13.5 ± 8.3	−4.0 ± 8.0	9.5	**<0**.001*	11.16	1.17
Torso lateral flexion (°)	6.8 ± 5.9	−4.2 ± 6.0	11.1	**<0**.001*	17.80	1.86
Torso rotation (°)	12.2 ± 10.2	−5.0 ± 10.9	17.2	**<0**.001*	15.56	1.63

Values are means ± SD. MD, mean difference.

* Significant mean difference (*p* ≤ 0.05). Torso flexion: positive values indicate torso forward flexion; torso lateral flexion: positive values indicate torso lateral flexion in the intended cutting direction; torso rotation: positive values indicate torso rotation in the intended cutting direction (bold used for visibility).

**Table 8:** The distribution of players between the two clusters in each injury group for the handball core 5 subset.

	Prev/New ACL group	Prev ACL group	New ACL group	No ACL group
Cluster 0 (*n* = 193)	1 (0.5%)	17 (8.8%)	10 (5.2%)	165 (85.5%)
Cluster 1 (*n* = 174)	1 (0.6%)	11 (6.3%)	10 (5.7%)	152 (87.4%)

Values are number of players (percentage of *n*); the Fisher-Freeman-Halton exact test yielded a *p*-value of 0.836; Prev/New ACL group, players with a previous ACL injury who went on to sustain a new secondary ACL injury; Prev ACL group, players with a previous ACL injury only; new ACL group, players without a previous ACL injury who went on to sustain a new primary ACL injury; no ACL group, injury free players.

**Figure 4:**
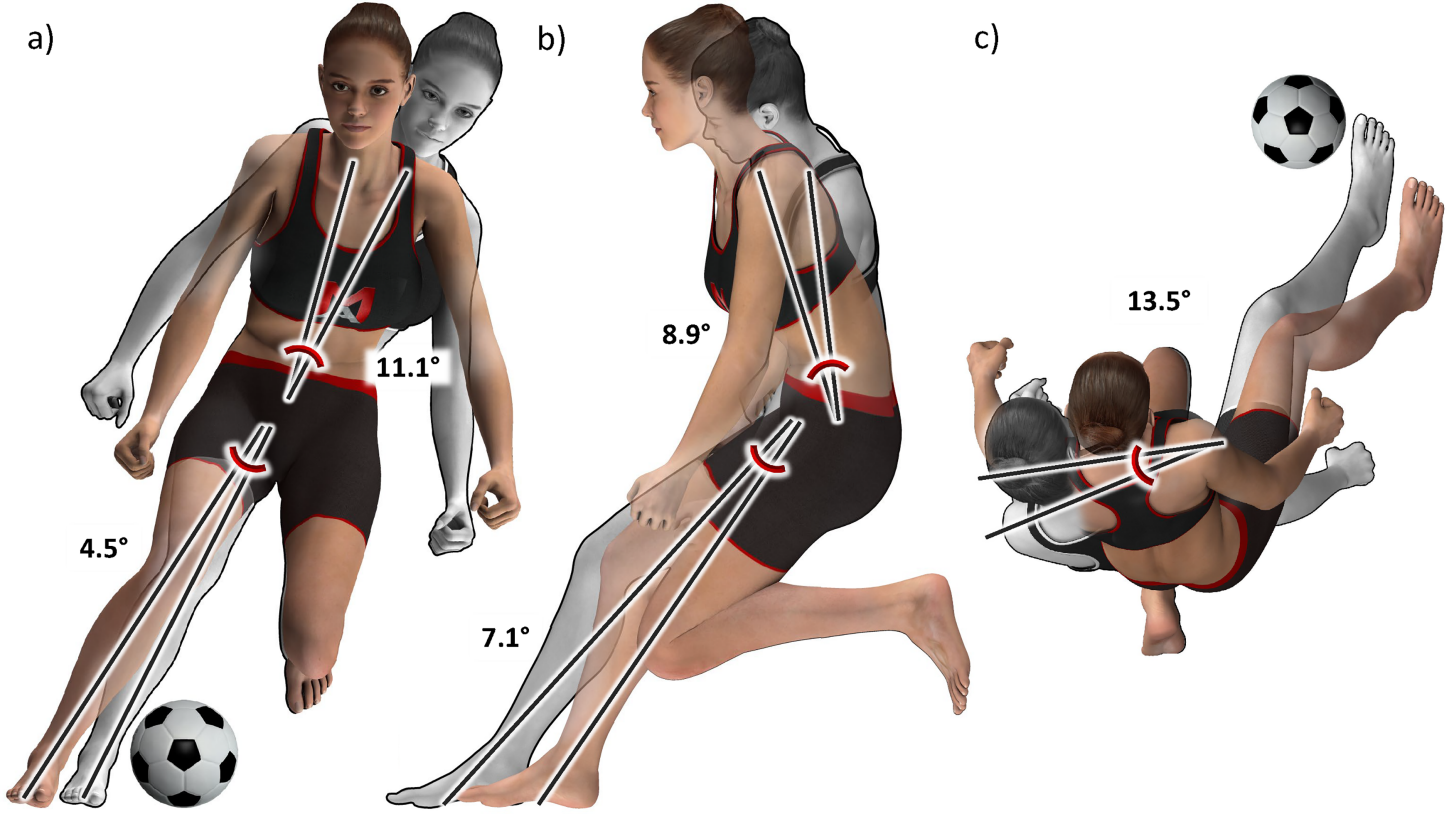
Animated figures for the Football Core 5 subset, illustrating the mean differences in cutting technique between Cluster 0 (grey) and Cluster 1 (color) in **(a)** the frontal plane, **(b)** the sagittal plane, and **(c)** the horizontal plane. The players nearest to the respective cluster centers, established by the Euclidean norm, were chosen as the basis. Created by Muscle Animations.

Descriptive and inferential statistics for the Football Core 5 subset are detailed in Table 9; Figure 4. Cutting depth was the input variable which separated the two identified cutting technique clusters the most, followed by torso lateral flexion and torso rotation (Table 9). The proportion of players did not differ between the two clusters (Fisher's *p* = 0. 472; Table 10), with players appearing randomly dispersed, as indicated by adjusted rand indices close to zero for the four injury groups and their binary derivates (Table 3). Notably, the peak knee abduction moments were significantly higher (*p* = 0.005; mean difference, 0.16 Nm/kg; Cohen's d, 0.29) in Cluster 1 (1.80 ± 0.59 Nm/kg) compared to Cluster 0 (1.64 ± 0.53 Nm/kg).

**Table 9:** Cluster descriptive and inferential statistics for the football core 5 subset.

	Cluster 0 (*n* = 161)	Cluster 1 (*n* = 219)	MD	*p*-value	Welch t-statistic	Cohen's d
Cutting width (°)	25.9 ± 4.2	30.4 ± 3.2	4.5	**<0**.001*	11.41	1.21
Cutting depth (°)	36.4 ± 3.2	29.3 ± 4.0	7.1	**<0**.001*	19.16	1.95
Torso flexion (°)	6.2 ± 9.9	15.2 ± 9.3	8.9	**<0**.001*	8.90	0.93
Torso lateral flexion (°)	19.6 ± 6.9	8.5 ± 6.4	11.1	**<0**.001*	15.95	1.67
Torso rotation (°)	19.5 ± 9.2	5.9 ± 8.1	13.5	**<0**.001*	14.87	1.56

Values are means ± SD. MD, mean difference.

* Significant mean difference (*p* ≤ 0.05). Torso flexion: positive values indicate torso forward flexion; torso lateral flexion: positive values indicate torso lateral flexion in the intended cutting direction; torso rotation: positive values indicate torso rotation in the intended cutting direction (bold used for visibility).

**Table 10:** The distribution of players between the two clusters in each injury group for the football core 5 subset.

	Prev/New ACL group	Prev ACL group	New ACL group	No ACL group
Cluster 0 (*n* = 161)	3 (1.9%)	9 (5.6%)	8 (5.0%)	141 (87.6%)
Cluster 1 (*n* = 219)	1 (0.5%)	9 (4.1%)	15 (6.8%)	194 (88.6%)

Values are number of players (percentage of *n*); the Fisher-Freeman-Halton exact test yielded a *p*-value of 0.472; Prev/New ACL group, players with a previous ACL injury who went on to sustain a new secondary ACL injury; Prev ACL group, players with a previous ACL injury only; new ACL group, players without a previous ACL injury who went on to sustain a new primary ACL injury; no ACL group, injury free players.

## 4 Discussion

This is the first study aiming to identify common cutting technique clusters and investigate their association with ACL injury risk in players with and without a previous ACL injury. We found no clearly distinguishable cutting technique clusters in the data set. There was however some evidence of cluster existence in the Core 5 subset involving both sports, but these cutting technique clusters were of no relevance for ACL injury risk. Rather, they could be attributed to sport- or task-related differences. We also found some evidence of cluster existence in the Football Core 5 subset. Even though peak knee abduction moments differed significantly between the identified clusters, no association with ACL injury risk could be found. The Handball Core 5 subset achieved the highest average silhouette score among the handball subsets. However, the identified cutting technique clusters were not associated with ACL injury risk and the peak knee abduction moments did not differ between the clusters. Hence, we could not identify specific movement strategies during cutting maneuvers clearly leading to a higher risk of primary or secondary ACL injury in this data set.

Not surprisingly, the average silhouette scores decreased with increasing numbers of clusters as well as with increasing number of variables included in the model (Table 2). This can be attributed to the “curse of dimensionality”, stating that an increase in dimensionality (i.e., variables) causes an exponential increase in the volume of the space which implies that the available data points become increasingly sparse, that data points belonging to the same cluster drift apart (i.e., the within-cluster distance increases) and that the distance between data points become more similar and less meaningful (43, 44). This makes it more difficult for the K-means algorithm to identify distinct and cohesive clusters and explains the decreasing average silhouette scores with added dimensions and clusters. To address this phenomenon, we included several subsets with fewer dimensions, and we applied PCA to reduce the number of dimensions computationally. Also, given this issue and the fact that the two cluster models often achieved the highest average silhouette score by a narrow margin, we chose to further explore the 3- and 4-cluster models as well. However, the respective results were unremarkable, and none of the identified cutting technique clusters proved to be relevant for ACL injury risk (Supplementary Material 2, 3).

### 4.1 Is cluster analysis useful for assessing ACL injury risk in a mixed group of handball and football players?

With an average silhouette score of 0.35, the Core 5 subset scored highest among all tested subsets (Table 2). Still, values between 0.25 and 0.5 are categorized only as medium evidence of cluster existence, and according to Larose and Larose (38) subject-specific expert opinion is required to determine if the differences between the clusters are meaningful. Players in Cluster 1 leaned 12° and rotated 8° more towards the intended cutting direction and they leaned 21° more forward than players in Cluster 0 (Table 4). They also placed their foot 8° further to the side and 7° further forward. All these five variables differed significantly between clusters and the effect sizes were “medium” to “huge” (45). Based on the mean differences, the effect sizes and the scatter plot visualization, we concluded that we have two distinguishable cutting technique clusters in this subset.

Still, the question remained whether or not these cutting technique clusters were of relevance for ACL injury risk. This did not appear to be the case. As can been seen in Table 5; Figure 2, the injured players appeared to be randomly distributed across the two clusters, with close to 50% of the players in each cluster. However, for the Prev/New ACL group, 5 out of 6 players were assigned to Cluster 1. Due to the small number of players in this group, the non-significant result of the Fisher-Freeman-Halton Exact test, and since we found a similar player distribution in the contralateral leg subset for this group (Supplementary Material 1), we suspect that this uneven distribution may be caused by chance. This is also confirmed by adjusted rand indices of zero, implying that the clustering results do not correspond with the injury groups. Therefore, the identified cutting technique clusters do not appear to be associated with ACL injury risk.

We were also interested to assess if the clustering results were affected by the two different sports which were included in this model. Since the cutting tasks were slightly different for the handball and football players, there was a chance that the algorithm would simply capture those differences. Not surprising, that is what we observed. Handball and football players were assigned to separate clusters (Table 6; Figure 2), which explains why the clustering results coincided well with the sport groups (Table 3). Therefore, the cutting technique clusters appeared to be based on sport-related differences rather than injury-related differences. Those differences in cutting technique between handball and football players were most likely caused by the slightly different cutting tasks rather than by sport-inherent differences. The handball players were instructed to fake- and pass a static defender, whereas the football players had to perform a sharp cut based on a football pass they received, without faking a defender.

On the one hand, these findings indicate that the clustering algorithm can successfully distinguish between genuinely distinct cutting techniques. At the same time, they indicate that the suggested thresholds for the average silhouette scores might be too conservative in the present context, given the low scores for the subsets involving both sports in spite of genuine differences between the clusters. On the other hand, the findings clearly indicate that we have to analyze different sports or slightly different tasks separately. Other biomechanical patterns which might be present in the data are likely to be washed out by these sport- or task-related patterns.

Despite the lack of association with ACL injury risk, the peak knee abduction moments were 13% higher (“small” effect size) in the cluster which was mainly represented by football players as compared to the “handball” cluster. This difference can likely be attributed to the wider cutting width in the “football” cluster (Table 4), which has previously been associated with larger knee abduction moments (29, 46). According to Kristianslund, Faul (29), increasing cutting width by 3.7° increases peak knee abduction moment by approximately 17%. In our study, the “football” cluster had an 8.1° wider cutting width yet only a 13% higher peak knee abduction moment. This can probably be explained by players in the “football” cluster leaning more in the direction of the cut (Table 4), which has been shown to reduce knee abduction moments (29, 46). The difference in peak knee abduction moments between the two clusters is likely attributable to the variations in the cutting tasks rather than inherent differences in the sports. This is further supported by previous research indicating similar ACL injury rates among football and handball players (18, 47).

### 4.2 Is cluster analysis useful for assessing ACL injury risk in handball players?

The Handball Core 5 subset scored highest among the handball subsets with an average silhouette score of 0.23 (Table 2). Since this score was close to the threshold of 0.25 and since we considered these thresholds as slightly conservative, we performed further analyses to assess the reality of the identified clusters. We found that players in Cluster 1 leaned 11° and rotated 17° more in the opposite direction of the cut, and they leaned 10° more forward than players in Cluster 0 (Table 7; Figure 3). The effect sizes for these torso variables were “large” to “very large”. The differences in cutting width and depth were negligible however with only 1–2° differences between clusters and “small” to “medium” effect sizes. Therefore, we concluded that there are medium distinct differences in cutting technique between those two clusters, warranting the assessment of those clusters' association with ACL injury risk.

The proportion of players with either a previous ACL injury or a new ACL injury during follow-up was similar in each identified cutting technique cluster (Table 8), implying that the risk of an ACL injury associated with these two different movement strategies is similar. This was also confirmed by adjusted rand indices near zero (Table 3), as well as by identical peak knee abduction moments across the clusters. Therefore, the differences in cutting technique between the two clusters appeared to have no practical relevance for ACL injury or ACL injury risk.

### 4.3 Is cluster analysis useful for assessing ACL injury risk in football players?

With an average silhouette score of 0.30, the Football Core 5 subset demonstrated some evidence of cluster existence (Table 2). We found that players in Cluster 1 leaned 11° and rotated 14° more in the opposite direction of the cut, and they leaned 9° more forward than players in Cluster 0 (Table 9; Figure 4). They also displayed a 5° wider cutting width and a 7° shorter cutting depth. All these five variables differed significantly between clusters and the effect sizes were “large” to “very large” (45). Based on the mean differences and the effect sizes, which were only slightly lower than those of the Core 5 subset (summated mean differences of 45.1° and 56.2°, respectively; summated mean effect sizes of 7.32 and 7.72, respectively), we concluded that we have two distinguishable cutting technique clusters in this subset.

The proportion of players with a previous ACL injury was similar in each identified cutting technique cluster (Tables 3, 10), implying that the clusters did not reflect the biomechanical alterations commonly observed in players with an ACL injury (48). The proportion of players who sustained a new ACL injury during follow-up was also similar across the two clusters, which indicates that the risk of an ACL injury associated with these two different movement strategies was similar.

Interestingly, the peak knee abduction moments were 10% higher (“small” effect size) in Cluster 1 compared to Cluster 0. This can likely be attributed to wider cutting widths in this cluster as well as more torso lateral flexion in the opposite direction of the cut (Figure 4), which both have previously been linked to greater knee abduction moments (29, 46). Prior research has shown that higher knee abduction moments result in higher ACL loading (49, 50) and may be associated with future ACL injury (17). Therefore, the increased peak knee abduction moments observed in Cluster 1 might potentially imply a higher risk of injury. However, since the difference and the effect size were small and since the injured players were distributed relatively evenly across these clusters, we can conclude that there is no clear evidence of an association between either of the cutting technique clusters and ACL injury risk.

### 4.4 Why are the identified clusters unrelated to ACL injury risk?

There are several possible explanations for the lack of association between the identified movement strategies and ACL injury risk. First, the cutting technique clusters may genuinely represent factors other than injury risk. For instance, they could be based on differences in genetics, anthropometry, muscular strength, coordination, playing position, or playing style. It is also conceivable that there might be a relationship to cutting performance, where one of the cutting strategies is more effective for outmaneuvering a defender than the other. Alternatively, these strategies could simply be random in nature with no specific underlying cause.

Second, the data may not clearly separate different cutting techniques relevant to injury, possibly due to insufficient information (e.g., not measuring or including relevant kinematic variables), measurement noise (e.g., inaccuracies in kinematic measurements), or a combination of both. Subtle differences in the motions might not have been captured because of the absence of additional biomechanical variables or the use of discrete rather than continuous data. Nonetheless, the clustering algorithm successfully distinguished between the two genuinely distinct cutting techniques in the Core 5 subset (i.e., handball vs. football cutting tasks), suggesting that the available information may have been sufficient.

Third, our laboratory task may not accurately reflect the biomechanics of game situations that lead to injury. This discrepancy could be due to the nature of the task itself, the level of effort exerted during its execution, or external factors such as additional cognitive demands.

### 4.5 Strengths and limitations

One notable strength of this study was its large sample size of 754 elite female athletes, including 59 with an ACL injury history and 56 who sustained an ACL injury during follow-up. These large numbers enhanced the chance of detecting commonly used cutting techniques among handball and football players and identifying their association with ACL injury risk. Still, there are some limitations to consider. First, we had a limited sample size of 13 players in the Prev/New ACL group, including 6 ACL re-injuries and 7 contralateral injuries. This complicated the detection of clear trends in the player distribution across clusters for this group. Second, while K-means stands out as a popular and efficient unsupervised machine learning algorithm which produces easily interpretable results, it does have its limitations (51). Notably, K-means assumes that clusters are spherical in shape and have similar sizes and densities (51), which could be a possible reason why this algorithm failed to identify clusters of relevance to ACL injury. To address more complex cluster shapes and sizes, future studies could explore alternative clustering algorithms like Gaussian mixture models, density-based methods, or hierarchical clustering. Finally, future research could consider incorporating kinetics into cluster analyses to potentially provide a deeper understanding of how certain combinations of loads could contribute to injury risk.

## 5 Conclusion

We identified two distinguishable cutting technique clusters in the subset involving both sports and 5 kinematics variables. However, these clusters were formed based on sport- or task-related differences rather than injury-related differences. Concordantly, the identified cutting technique clusters in the handball and football subsets with 5 kinematic variables were also found to be unrelated to ACL injury risk.

Overall, K-means cluster analysis methodology proved valuable for identifying different cutting techniques. However, none of the identified cutting techniques seemed to increase the risk of ACL injury, implying that we could not identify safe or risky side-step cutting technique strategies among our cohort of 754 female elite handball and football players. Therefore, cluster analysis of cutting technique, using a K-means algorithm, did not prove to be a valuable approach for assessing ACL injury risk in this dataset.

## Data Availability

The raw data supporting the conclusions of this article will be made available by the authors, without undue reservation.

## References

[1] Chia L De Oliveira Silva D Whalan M McKay MJ Sullivan J Fuller CW Non-contact anterior cruciate ligament injury epidemiology in team-ball sports: a systematic review with meta-analysis by sex, age, sport, participation level, and exposure type. Sports Med. (2022) 52(10):2447–67. 10.1007/s40279-022-01697-w 35622227 PMC9136558

[2] Wiggins AJ Grandhi RK Schneider DK Stanfield D Webster KE Myer GD. Risk of secondary injury in younger athletes after anterior cruciate ligament reconstruction: a systematic review and meta-analysis. Am J Sports Med. (2016) 44(7):1861–76. 10.1177/0363546515621554 26772611 PMC5501245

[3] Grassi A Zaffagnini S Marcheggiani Muccioli GM Neri MP Della Villa S Marcacci M. After revision anterior cruciate ligament reconstruction, who returns to sport? A systematic review and meta-analysis. Br J Sports Med. (2015) 49(20):1295–304. 10.1136/bjsports-2014-094089 26062956

[4] Fältström A Hägglund M Kvist J. Patient-reported knee function, quality of life, and activity level after bilateral anterior cruciate ligament injuries. Am J Sports Med. (2013) 41(12):2805–13. 10.1177/0363546513502309 24007758

[5] Grassi A Ardern CL Marcheggiani Muccioli GM Neri MP Marcacci M Zaffagnini S. Does revision ACL reconstruction measure up to primary surgery? A meta-analysis comparing patient-reported and clinician-reported outcomes, and radiographic results. Br J Sports Med. (2016) 50(12):716–24. 10.1136/bjsports-2015-094948 26809259

[6] Filbay SR Ackerman IN Russell TG Macri EM Crossley KM. Health-related quality of life after anterior cruciate ligament reconstruction: a systematic review. Am J Sports Med. (2014) 42(5):1247–55. 10.1177/0363546513512774 24318609

[7] Bahr R Krosshaug T. Understanding injury mechanisms: a key component of preventing injuries in sport. Br J Sports Med. (2005) 39(6):324. 10.1136/bjsm.2005.018341 15911600 PMC1725226

[8] Alentorn-Geli E Myer GD Silvers HJ Samitier G Romero D Lázaro-Haro C Prevention of non-contact anterior cruciate ligament injuries in soccer players. Part 1: mechanisms of injury and underlying risk factors. Knee Surg Sports Traumatol Arthrosc. (2009) 17(7):705–29. 10.1007/s00167-009-0813-1 19452139

[9] Anderson MJ Browning WM 3rd Urband CE Kluczynski MA Bisson LJ. A systematic summary of systematic reviews on the topic of the anterior cruciate ligament. Orthop J Sports Med. (2016) 4(3):2325967116634074. 10.1177/2325967116634074 27047983 PMC4794976

[10] Cronström A Tengman E Häger CK. Risk factors for contra-lateral secondary anterior cruciate ligament injury: a systematic review with meta-analysis. Sports Med. (2021) 51(7):1419–38. 10.1007/s40279-020-01424-3 33515391 PMC8222029

[11] Cronström A Tengman E Häger CK. Return to sports: a risky business? A systematic review with meta-analysis of risk factors for graft rupture following ACL reconstruction. Sports Med. (2023) 53(1):91–110. 10.1007/s40279-022-01747-3 36001289 PMC9807539

[12] Hewett TE Di Stasi SL Myer GD. Current concepts for injury prevention in athletes after anterior cruciate ligament reconstruction. Am J Sports Med. (2013) 41(1):216–24. 10.1177/0363546512459638 23041233 PMC3592333

[13] Myklebust G Maehlum S Engebretsen L Strand T Solheim E. Registration of cruciate ligament injuries in Norwegian top level team handball. A prospective study covering two seasons. Scand J Med Sci Sports. (1997) 7(5):289–92. 10.1111/j.1600-0838.1997.tb00155.x 9338947

[14] Myklebust G Maehlum S Holm I Bahr R. A prospective cohort study of anterior cruciate ligament injuries in elite Norwegian team handball. Scand J Med Sci Sports. (1998) 8(3):149–53. 10.1111/j.1600-0838.1998.tb00185.x 9659675

[15] Faunø P Wulff Jakobsen B. Mechanism of anterior cruciate ligament injuries in soccer. Int J Sports Med. (2006) 27(1):75–9. 10.1055/s-2005-837485 16388446

[16] Francesco Della V Matthew B Alberto G Alberto N Filippo T Stefano Z Systematic video analysis of ACL injuries in professional male football (soccer): injury mechanisms, situational patterns and biomechanics study on 134 consecutive cases. Br J Sports Med. (2020) 54(23):1423. 10.1136/bjsports-2019-101247 32561515

[17] Hewett TE Myer GD Ford KR Heidt RS Jr. Colosimo AJ McLean SG Biomechanical measures of neuromuscular control and valgus loading of the knee predict anterior cruciate ligament injury risk in female athletes: a prospective study. Am J Sports Med. (2005) 33(4):492–501. 10.1177/0363546504269591 15722287

[18] Krosshaug T Steffen K Kristianslund E Nilstad A Mok KM Myklebust G The vertical drop jump is a poor screening test for ACL injuries in female elite soccer and handball players: a prospective cohort study of 710 athletes. Am J Sports Med. (2016) 44(4):874–83. 10.1177/0363546515625048 26867936

[19] Leppänen M Pasanen K Kujala UM Vasankari T Kannus P Äyrämö S Stiff landings are associated with increased ACL injury risk in young female basketball and floorball players. Am J Sports Med. (2017) 45(2):386–93. 10.1177/0363546516665810 27637264

[20] Leppänen M Pasanen K Krosshaug T Kannus P Vasankari T Kujala UM Sagittal plane hip, knee, and ankle biomechanics and the risk of anterior cruciate ligament injury: a prospective study. Orthop J Sports Med. (2017) 5(12):2325967117745487. 10.1177/2325967117745487 29318174 PMC5753918

[21] Cronström A Creaby MW Ageberg E. Do knee abduction kinematics and kinetics predict future anterior cruciate ligament injury risk? A systematic review and meta-analysis of prospective studies. BMC Musculoskelet Disord. (2020) 21(1):563. 10.1186/s12891-020-03552-3 32819327 PMC7441716

[22] Hewett TE Ford KR Xu YY Khoury J Myer GD. Utilization of ACL injury biomechanical and neuromuscular risk profile analysis to determine the effectiveness of neuromuscular training. Am J Sports Med. (2016) 44(12):3146–51. 10.1177/0363546516656373 27474385 PMC5513480

[23] Bird MB Mi Q Koltun KJ Lovalekar M Martin BJ Fain A Unsupervised clustering techniques identify movement strategies in the countermovement jump associated with musculoskeletal injury risk during US marine corps officer candidates school. Front Physiol. (2022) 13:1–14. 10.3389/fphys.2022.868002 PMC9132209 35634154

[24] Steffen K Nilstad A Kristianslund EK Myklebust G Bahr R Krosshaug T. Association between lower extremity muscle strength and noncontact ACL injuries. Med Sci Sports Exerc. (2016) 48(11):2082–9. 10.1249/MSS.0000000000001014 27327027

[25] Nilstad A Petushek E Mok KM Bahr R Krosshaug T. Kiss goodbye to the “kissing knees”: no association between frontal plane inward knee motion and risk of future non-contact ACL injury in elite female athletes. Sports Biomech. (2023) 22(1):65–79. 10.1080/14763141.2021.1903541 33906580

[26] Petushek E Nilstad A Bahr R Krosshaug T. Drop jump? Single-leg squat? Not if you aim to predict anterior cruciate ligament injury from real-time clinical assessment: a prospective cohort study involving 880 elite female athletes. J Orthop Sports Phys Ther. (2021) 51(7):372–8. 10.2519/jospt.2021.10170 34192883

[27] Mok KM Bahr R Krosshaug T. Reliability of lower limb biomechanics in two sport-specific sidestep cutting tasks. Sports Biomech. (2018) 17(2):157–67. 10.1080/14763141.2016.1260766 28281390

[28] Kristianslund E Krosshaug T van den Bogert AJ. Effect of low pass filtering on joint moments from inverse dynamics: implications for injury prevention. J Biomech. (2012) 45(4):666–71. 10.1016/j.jbiomech.2011.12.011 22227316

[29] Kristianslund E Faul O Bahr R Myklebust G Krosshaug T. Sidestep cutting technique and knee abduction loading: implications for ACL prevention exercises. Br J Sports Med. (2014) 48(9):779–83. 10.1136/bjsports-2012-091370 23258848

[30] Paterno MV Schmitt LC Ford KR Rauh MJ Myer GD Huang B Biomechanical measures during landing and postural stability predict second anterior cruciate ligament injury after anterior cruciate ligament reconstruction and return to sport. Am J Sports Med. (2010) 38(10):1968–78. 10.1177/0363546510376053 20702858 PMC4920967

[31] Koga H Nakamae A Shima Y Iwasa J Myklebust G Engebretsen L Mechanisms for noncontact anterior cruciate ligament injuries: knee joint kinematics in 10 injury situations from female team handball and basketball. Am J Sports Med. (2010) 38(11):2218–25. 10.1177/0363546510373570 20595545

[32] King E Richter C Daniels KAJ Franklyn-Miller A Falvey E Myer GD Can biomechanical testing after anterior cruciate ligament reconstruction identify athletes at risk for subsequent ACL injury to the contralateral uninjured limb? Am J Sports Med. (2021) 49(3):609–19. 10.1177/0363546520985283 33560866 PMC9938948

[33] King E Richter C Daniels KAJ Franklyn-Miller A Falvey E Myer GD Biomechanical but not strength or performance measures differentiate male athletes who experience ACL reinjury on return to level 1 sports. Am J Sports Med. (2021) 49(4):918–27. 10.1177/0363546520988018 33617291 PMC9677345

[34] Arthur D Vassilvitskii S. K-means++: the advantages of careful seeding. Conference: Proceedings of the Eighteenth Annual ACM-SIAM Symposium on Discrete Algorithms, SODA 2007; 2007 Jan 7–9; New Orleans, Louisiana, USA. Philadelphia, PA: Society for Industrial and Applied Mathematics (2007). 10.1145/1283383.1283494

[35] Singh A Yadav A Rana A. K-means with three different distance metrics. Int J Comput Appl. (2013) 67:13–7. 10.5120/11430-6785

[36] Danny Matthew S Daniel S Liniyanti DO. Effect of distance metrics in determining K-value in K-means clustering using elbow and silhouette method. Proceedings of the Sriwijaya International Conference on Information Technology and Its Applications (SICONIAN 2019); 2020/05/06; Atlantis Press. (2020).

[37] Rousseeuw PJ. Silhouettes: a graphical aid to the interpretation and validation of cluster analysis. J Comput Appl Math. (1987) 20:53–65. 10.1016/0377-0427(87)90125-7

[38] Larose DT Larose CD. Data Mining and Predictive Analytics. 2nd ed. Hoboken, NJ: John Wiley & Sons, Inc. (2015).

[39] Rand WM. Objective criteria for the evaluation of clustering methods. J Am Stat Assoc. (1971) 66(336):846–50. 10.1080/01621459.1971.10482356

[40] Hubert L Arabie P. Comparing partitions. J Classif. (1985) 2(1):193–218. 10.1007/BF01908075

[41] Jauhiainen S Pohl AJ Äyrämö S Kauppi J-P Ferber R. A hierarchical cluster analysis to determine whether injured runners exhibit similar kinematic gait patterns. Scand J Med Sci Sports. (2020) 30(4):732–40. 10.1111/sms.13624 31900980

[42] Hewett TE Ford KR Hoogenboom BJ Myer GD. Understanding and preventing ACL injuries: current biomechanical and epidemiologic considerations—update 2010. N Am J Sports Phys Ther. (2010) 5(4):234–51. 21655382 PMC3096145

[43] Molchanov V Linsen L. Overcoming the curse of dimensionality when clustering multivariate volume data. In Proceedings of the 13th International Joint Conference on Computer Vision, Imaging and Computer Graphics Theory and Applications (VISIGRAPP 2018)- Volume 3: IVAPP. Setúbal: SciTePress (2018). p. 29–39. 10.5220/0006541900290039

[44] Steinbach M Ertöz L Kumar V. The challenges of clustering high dimensional data. Univ Minnesota Supercomp Inst Res Rep. (2004) 213. 10.1007/978-3-662-08968-2_16

[45] Sawilowsky S. New effect size rules of thumb. J Mod Appl Stat Methods. (2009) 8:597–9. 10.22237/jmasm/1257035100

[46] Dempsey AR Lloyd DG Elliott BC Steele JR Munro BJ Russo KA. The effect of technique change on knee loads during sidestep cutting. Med Sci Sports Exerc. (2007) 39(10):1765–73. 10.1249/mss.0b013e31812f56d1 17909404

[47] Michaelidis M Koumantakis GA. Effects of knee injury primary prevention programs on anterior cruciate ligament injury rates in female athletes in different sports: a systematic review. Phys Ther Sport. (2014) 15(3):200–10. 10.1016/j.ptsp.2013.12.002 24703497

[48] Mausehund L Krosshaug T. Knee biomechanics during cutting maneuvers and secondary ACL injury risk: a prospective cohort study of knee biomechanics in 756 female elite handball and soccer players. Am J Sports Med. (2024) 52(5):1209–19. 10.1177/03635465241234255 38459717 PMC10986153

[49] Fukuda Y Woo SL Loh JC Tsuda E Tang P McMahon PJ A quantitative analysis of valgus torque on the ACL: a human cadaveric study. J Orthop Res. (2003) 21(6):1107–12. 10.1016/S0736-0266(03)00084-6 14554225

[50] Shin CS Chaudhari AM Andriacchi TP. Valgus plus internal rotation moments increase anterior cruciate ligament strain more than either alone. Med Sci Sports Exerc. (2011) 43(8):1484–91. 10.1249/MSS.0b013e31820f8395 21266934

[51] Raykov YP Boukouvalas A Baig F Little MA. What to do when K-means clustering fails: a simple yet principled alternative algorithm. PLoS One. (2016) 11(9):e0162259. 10.1371/journal.pone.0162259 27669525 PMC5036949

