# Supplementary material for: Cluster analysis of cutting technique—a valuable approach for assessing anterior cruciate ligament injury risk?
Source: Front Sports Act Living. 2025 Feb 10;7:1463272. doi: 10.3389/fspor.2025.1463272 (PMC11847870; doi:10.3389/fspor.2025.1463272)
Supplement: Supplementary file 1 [file Datasheet1.pdf]

Supplementary Material

1. Results of the contralateral leg subsets

Supplementary Table 1. *Average silhouette scores for all subsets and for different number of clusters.*

	2 clusters	3 clusters	4 clusters	5 clusters
**All 36**	**0.16***	0.14	0.13	0.11
**All 36 (PCA)**	**0.19***	0.16	0.14	0.13
**Core 13**	**0.23***	0.18	0.16	0.15
**Core 5**	**0.35*, ‡**	0.30‡	0.25‡	0.21
**Handball All 36**	**0.12***	0.09	0.08	0.07
**Handball All 36 (PCA)**	**0.12***	0.11	0.11	0.09
**Handball Core 13**	**0.14***	0.13	0.12	0.11
**Handball Core 5**	**0.23*, ‡**	0.20	0.16	0.17
**Football All 36**	**0.14***	0.10	0.08	0.07
**Football All 36 (PCA)**	**0.15***	0.12	0.10	0.10
**Football Core 13**	**0.19***	0.11	0.11	0.11
**Football Core 5**	**0.30*, ‡**	0.21	0.20	0.19

* The highest average silhouette score of all cluster models within the same subset.
‡ Subsets with an average silhouette score exceeding 0.25, indicating some evidence of cluster existence, and subsets scoring highest within their sport group.
The first four subsets involve both handball and football players as well as a selection of kinematic variables (all 36 variables, a narrowed selection of 13 and 5 variables, and a reduction using principal component analysis, PCA). The last eight subsets involve either handball or football players alone and the same selection of variables.

Supplementary Table 2. *Cluster descriptive and inferential statistics for the Core 5 subset.*

	Cluster 0 (n = 375)	Cluster 1 (n = 376)	MD	p-value	Welch t-statistic	Cohen's d
**Cutting width (°)**	28.6 ± 4.3	20.5 ± 3.8	8.1	**<0.001***	27.05	1.97
**Cutting depth (°)**	32.4 ± 5.0	25.8 ± 4.6	6.5	**<0.001***	18.45	1.35
**Torso flexion (°)**	11.5 ± 10.1	-9.2 ± 9.3	20.7	**<0.001***	29.22	2.13
**Torso lateral flexion (°)**	13.6 ± 8.1	1.1 ± 7.7	12.5	**<0.001***	21.73	1.59
**Torso rotation (°)**	12.6 ± 10.8	3.6 ± 13.5	9.0	**<0.001***	10.08	0.74

Values are means ± SD. MD, mean difference; *significant mean difference ($p \leq 0.05$). Torso flexion: Positive values indicate torso forward flexion; Torso lateral flexion: Positive values indicate torso lateral flexion in the intended cutting direction; Torso rotation: Positive values indicate torso rotation in the intended cutting direction.

Supplementary Table 3. *The distribution of players between the two clusters in each injury group for the Core 5 subset.*

	Prev/New ACL group	Prev ACL group	New ACL group	No ACL group
**Cluster 0 (n = 375)**	3 (0.8%)	20 (5.3%)	22 (5.9%)	330 (88.0%)
**Cluster 1 (n = 376)**	4 (1.1%)	29 (7.7%)	21 (5.6%)	322 (85.6%)

Values are number of players (percentage of n); The Fisher-Freeman-Halton Exact test yielded a p-value of 0.589; Prev/New ACL group, players with a previous ACL injury who went on to sustain a new secondary ACL injury; Prev ACL group, players with a previous ACL injury only; New ACL group, players without a previous ACL injury who went on to sustain a new primary ACL injury; No ACL group, injury free players.

Supplementary Table 4. *The distribution of players between the two clusters in each sport group for the Core 5 subset.*

	Football	Handball
**Cluster 0 (n = 375)**	363 (96.8%)	12 (3.2%)
**Cluster 1 (n = 376)**	17 (4.5%)	359 (95.5%)

Values are number of players (percentage of n); The Fisher-Freeman-Halton Exact test yielded a p-value of < 0.001.

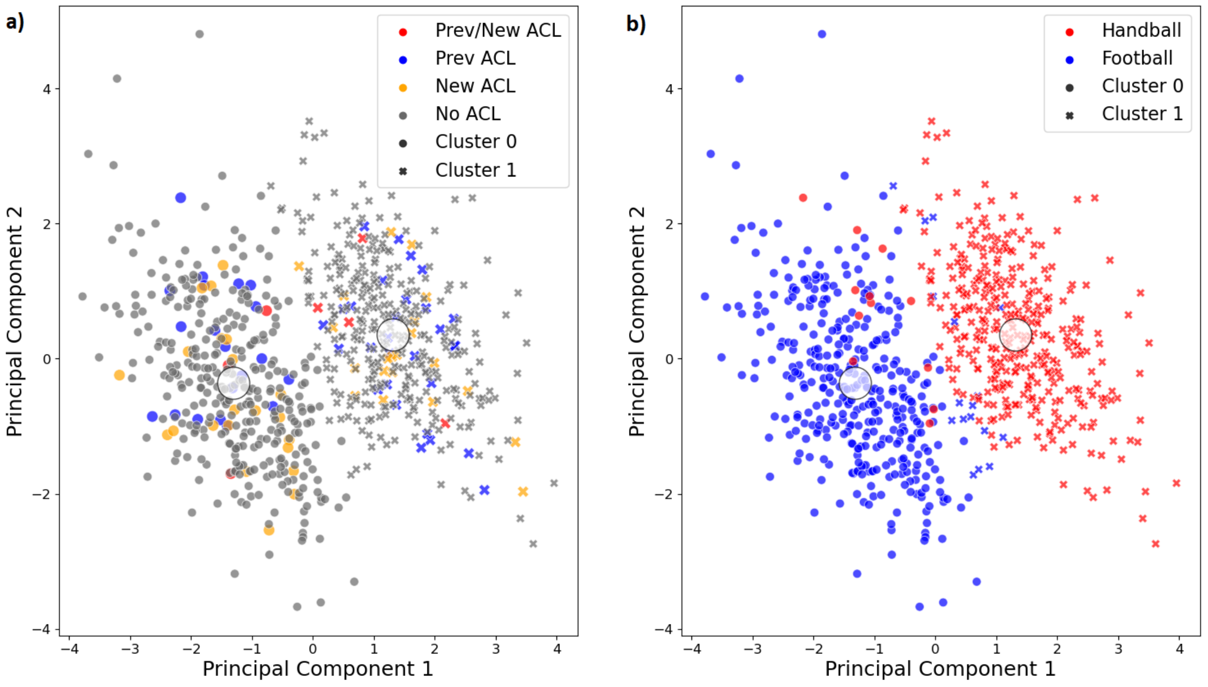

Supplementary Figure 1*. Visualization of the two clusters (Cluster 0 and Cluster 1) as well as the true labels of* ***(A)*** *the injury groups and* ***(B)*** *the sport groups for the Core 5 subset. The first two principal components of the principal component analysis, which explain the highest amount of variance in the original data, are plotted against each other. Prev/New ACL group, players with a previous ACL injury who went on to sustain a new secondary ACL injury; Prev ACL group, players with a previous ACL injury only; New ACL group, players without a previous ACL injury who went on to sustain a new primary ACL injury; No ACL group, injury free players.*

Supplementary Table 5. *Cluster descriptive and inferential statistics for the Handball Core 5 subset.*

	Cluster 0 (n = 186)	Cluster 1 (n = 185)	MD	p-value	Welch t-statistic	Cohen's d
**Cutting width (°)**	21.0 ± 3.3	19.9 ± 4.1	1.1	**0.007***	2.73	0.28
**Cutting depth (°)**	25.1 ± 4.5	26.7 ± 4.8	1.6	**0.001***	3.38	0.35
**Torso flexion (°)**	-3.8 ± 7.2	-14.5 ± 8.0	10.7	**<0.001***	13.48	1.40
**Torso lateral flexion (°)**	-4.1 ± 5.9	6.9 ± 5.7	11.0	**<0.001***	18.35	1.91
**Torso rotation (°)**	-4.0 ± 11.8	12.2 ± 10.6	16.2	**<0.001***	13.93	1.45

Values are means ± SD. MD, mean difference; *significant mean difference ($p \leq 0.05$). Torso flexion: Positive values indicate torso forward flexion; Torso lateral flexion: Positive values indicate torso lateral flexion in the intended cutting direction; Torso rotation: Positive values indicate torso rotation in the intended cutting direction.

Supplementary Table 6. *The distribution of players between the two clusters in each injury group for the Handball Core 5 subset.*

	Prev/New ACL group	Prev ACL group	New ACL group	No ACL group
**Cluster 0 (n = 186)**	1 (0.5%)	17 (9.1%)	10 (5.4%)	158 (84.9%)
**Cluster 1 (n = 185)**	3 (1.6%)	13 (7.0%)	10 (5.4%)	159 (85.9%)

Values are number of players (percentage of n); The Fisher-Freeman-Halton Exact test yielded a p-value of 0.702; Prev/New ACL group, players with a previous ACL injury who went on to sustain a new secondary ACL injury; Prev ACL group, players with a previous ACL injury only; New ACL group, players without a previous ACL injury who went on to sustain a new primary ACL injury; No ACL group, injury free players.

Supplementary Table 7. *Cluster descriptive and inferential statistics for the Football Core 5 subset.*

	Cluster 0 (n = 166)	Cluster 1 (n = 214)	MD	p-value	Welch t-statistic	Cohen's d
**Cutting width (°)**	25.8 ± 4.1	30.7 ± 3.0	4.9	**<0.001***	13.02	1.37
**Cutting depth (°)**	36.2 ± 3.3	29.2 ± 4.0	7.0	**<0.001***	18.74	1.91
**Torso flexion (°)**	6.0 ± 9.8	15.2 ± 9.3	9.2	**<0.001***	9.28	0.96
**Torso lateral flexion (°)**	18.9 ± 6.8	8.7 ± 6.5	10.3	**<0.001***	14.91	1.55
**Torso rotation (°)**	19.3 ± 9.3	6.2 ± 8.2	13.1	**<0.001***	14.25	1.49

Values are means ± SD. MD, mean difference; *significant mean difference ($p \leq 0.05$). Torso flexion: Positive values indicate torso forward flexion; Torso lateral flexion: Positive values indicate torso lateral flexion in the intended cutting direction; Torso rotation: Positive values indicate torso rotation in the intended cutting direction.

Supplementary Table 8. *The distribution of players between the two clusters in each injury group for the Football Core 5 subset.*

	Prev/New ACL group	Prev ACL group	New ACL group	No ACL group
**Cluster 0 (n = 166)**	2 (1.2%)	10 (6.0%)	8 (4.8%)	146 (88.0%)
**Cluster 1 (n = 214)**	1 (0.5%)	9 (4.2%)	15 (7.0%)	189 (88.3%)

Values are number of players (percentage of n); The Fisher-Freeman-Halton Exact test yielded a p-value of 0.576; Prev/New ACL group, players with a previous ACL injury who went on to sustain a new secondary ACL injury; Prev ACL group, players with a previous ACL injury only; New ACL group, players without a previous ACL injury who went on to sustain a new primary ACL injury; No ACL group, injury free players.

Supplementary Table 9. *Adjusted rand indices comparing the clustering results with the true labels of four different injury groupings and one sport grouping.*

	Injury groups				Sport groups
		BIN No ACL group	***BIN Prev ACL group***	***BIN New ACL group***	
**Core 5**	0.00	0.00	0.00	0.00	0.85
**Handball Core 5**	0.00	0.00	0.00	0.00	n.a.
**Football Core 5**	0.00	0.00	0.01	0.00	n.a.

Injury groups, the four injury groups depicted in Figure 1 (main article); BIN No ACL group, No ACL group vs. all other groups; BIN Prev ACL group, Prev/New ACL group and Prev ACL group vs. all other groups; BIN New ACL group, Prev/New ACL group and New ACL group vs. all other groups; Sport groups, Handball vs. football players.

2. Results for the 3-cluster models

Supplementary Table 10. *Cluster descriptive statistics for the Core 5 subset.*

		Cutting width (°)	Cutting depth (°)	Torso flexion (°)	Torso lateral flexion (°)	Torso rotation (°)
**Ipsilateral leg subset**	**Cluster 0 (n = 192)**	26.4 ± 4.2	36.0 ± 3.3	6.9 ± 9.7	18.8 ± 6.9	18.4 ± 9.6
	**Cluster 1 (n = 356)**	20.3 ± 3.7	25.8 ± 4.6	-9.6 ± 8.9	1.2 ± 7.8	3.7 ± 13.4
	**Cluster 2 (n = 199)**	30.4 ± 3.3	28.7 ± 3.7	15.7 ± 9.1	7.8 ± 6.3	5.5 ± 8.1
**Contralateral leg subset**	**Cluster 0 (n = 368)**	20.5 ± 3.8	25.7 ± 4.5	-9.2 ± 9.1	1.0 ± 7.6	3.4 ± 13.5
	**Cluster 1 (n = 198)**	30.7 ± 3.1	28.9 ± 3.9	16.6 ± 8.5	8.8 ± 6.5	6.5 ± 8.3
	**Cluster 2 (n = 185)**	26.1 ± 4.3	36.0 ± 3.3	5.2 ± 9.6	18.5 ± 6.6	19.1 ± 9.4

Values are means ± SD. Torso flexion: Positive values indicate torso forward flexion; Torso lateral flexion: Positive values indicate torso lateral flexion in the intended cutting direction; Torso rotation: Positive values indicate torso rotation in the intended cutting direction.

Supplementary Table 11. *The distribution of players between the three clusters in each injury group for the Core 5 subset.*

		Prev/New ACL group	Prev ACL group	New ACL group	No ACL group
**Ipsilateral leg subset**	**Cluster 0 (n = 192)**	4 (2.1%)	13 (6.8%)	9 (4.7%)	166 (86.5%)
	**Cluster 1 (n = 356)**	1 (0.3%)	25 (7.0%)	21 (5.9%)	309 (86.8%)
	**Cluster 2 (n = 199)**	1 (0.5%)	8 (4.0%)	13 (6.5%)	177 (88.9%)
**Contralateral leg subset**	**Cluster 0 (n = 368)**	4 (1.1%)	29 (7.9%)	21 (5.7%)	314 (85.3%)
	**Cluster 1 (n = 198)**	1 (0.5%)	8 (4.0%)	14 (7.1%)	175 (88.4%)
	**Cluster 2 (n = 185)**	2 (1.1%)	12 (6.5%)	8 (4.3%)	163 (88.1%)

Values are number of players (percentage of n); The Fisher-Freeman-Halton Exact test yielded a p-value of 0.287 and 0.558 for the ipsilateral and contralateral subset, respectively; Prev/New ACL group, players with a previous ACL injury who went on to sustain a new secondary ACL injury; Prev ACL group, players with a previous ACL injury only; New ACL group, players without a previous ACL injury who went on to sustain a new primary ACL injury; No ACL group, injury free players.

Supplementary Table 12. *The distribution of players between the three clusters in each sport group for the Core 5 subset.*

		Football	Handball
**Ipsilateral leg subset**	**Cluster 0 (n = 192)**	181 (94.3%)	11 (5.7%)
	**Cluster 1 (n = 356)**	8 (2.2%)	348 (97.8%)
	**Cluster 2 (n = 199)**	191 (96.0%)	8 (4.0%)
**Contralateral leg subset**	**Cluster 0 (n = 368)**	13 (3.5%)	355 (96.5%)
	**Cluster 1 (n = 198)**	194 (98.0%)	4 (2.0%)
	**Cluster 2 (n = 185)**	173 (93.5%)	12 (6.5%)

Values are number of players (percentage of n); The Fisher-Freeman-Halton Exact test yielded a p-value of < 0.001 and < 0.001 for the ipsilateral and contralateral subset, respectively.

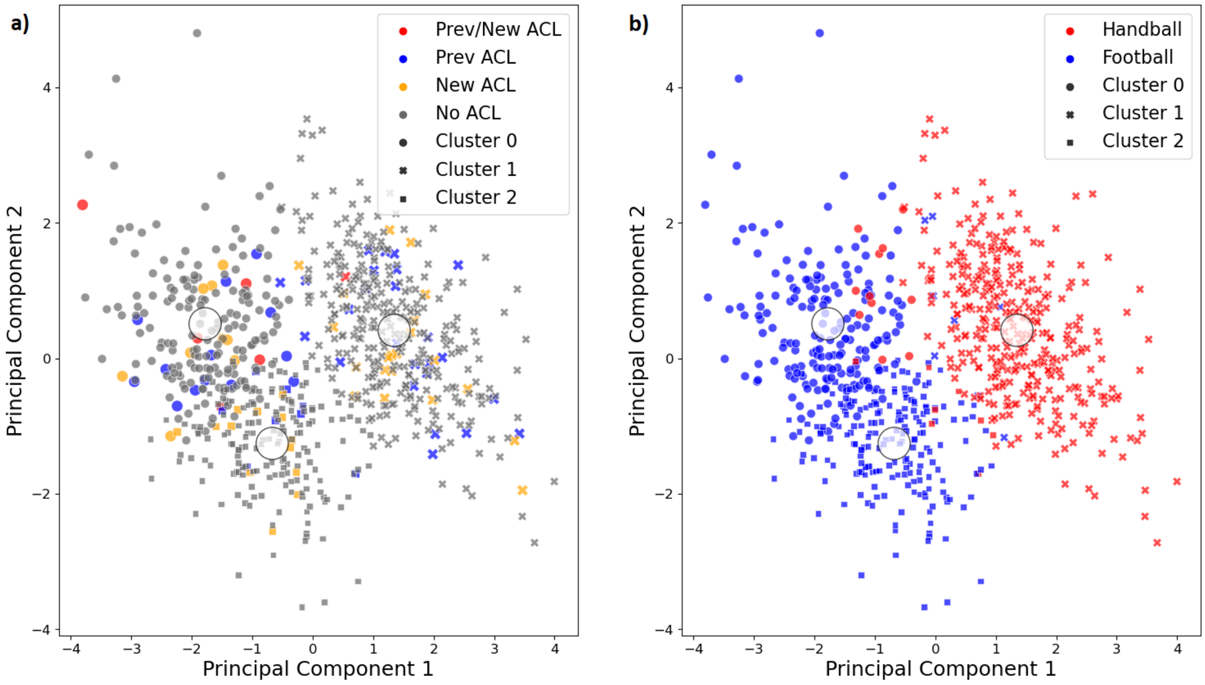

Supplementary Figure 2*. Visualization of the three clusters (Cluster 0, Cluster 1 and Cluster 2) as well as the true labels of* ***(A)*** *the injury groups and* ***(B)*** *the sport groups for the Core 5 subset (ipsilateral leg subset). The first two principal components of the principal component analysis, which explain the highest amount of variance in the original data, are plotted against each other. Prev/New ACL group, players with a previous ACL injury who went on to sustain a new secondary ACL injury; Prev ACL group, players with a previous ACL injury only; New ACL group, players without a previous ACL injury who went on to sustain a new primary ACL injury; No ACL group, injury free players.*

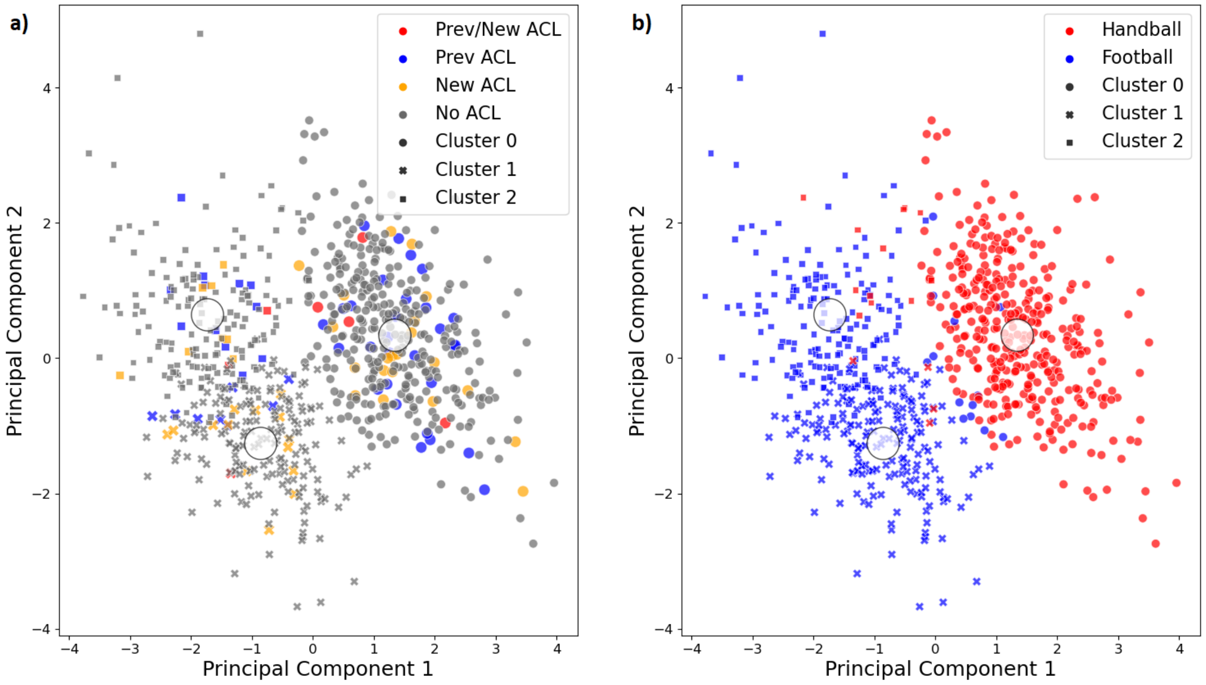

Supplementary Figure 3*. Visualization of the three clusters (Cluster 0, Cluster 1 and Cluster 2) as well as the true labels of* ***(A)*** *the injury groups and* ***(B)*** *the sport groups for the Core 5 subset (contralateral leg subset). The first two principal components of the principal component analysis, which explain the highest amount of variance in the original data, are plotted against each other. Prev/New ACL group, players with a previous ACL injury who went on to sustain a new secondary ACL injury; Prev ACL group, players with a previous ACL injury only; New ACL group, players without a previous ACL injury who went on to sustain a new primary ACL injury; No ACL group, injury free players.*

Supplementary Table 13. *Cluster descriptive statistics for the Handball Core 5 subset.*

		Cutting width (°)	Cutting depth (°)	Torso flexion (°)	Torso lateral flexion (°)	Torso rotation (°)
**Ipsilateral leg subset**	**Cluster 0 (n = 105)**	22.1 ± 3.5	22.6 ± 3.7	-16.2 ± 7.1	5.1 ± 6.0	4.8 ± 9.9
	**Cluster 1 (n = 139)**	21.0 ± 3.2	25.2 ± 4.2	-2.1 ± 6.6	-5.3 ± 5.9	-5.5 ± 11.2
	**Cluster 2 (n = 123)**	18.3 ± 3.8	29.6 ± 3.2	-10.7 ± 8.4	6.4 ± 6.3	14.3 ± 10.8
**Contralateral leg subset**	**Cluster 0 (n = 109)**	22.0 ± 3.5	22.5 ± 3.7	-16.4 ± 7.3	5.1 ± 5.8	5.8 ± 9.9
	**Cluster 1 (n = 120)**	18.4 ± 3.8	29.8 ± 3.3	-11.1 ± 7.6	5.8 ± 6.6	13.8 ± 11.4
	**Cluster 2 (n = 142)**	21.0 ± 3.2	25.2 ± 4.0	-1.9 ± 6.3	-5.1 ± 5.9	-5.5 ± 11.8

Values are means ± SD. Torso flexion: Positive values indicate torso forward flexion; Torso lateral flexion: Positive values indicate torso lateral flexion in the intended cutting direction; Torso rotation: Positive values indicate torso rotation in the intended cutting direction.

Supplementary Table 14. *The distribution of players between the three clusters in each injury group for the Handball Core 5 subset.*

		Prev/New ACL group	Prev ACL group	New ACL group	No ACL group
**Ipsilateral leg subset**	**Cluster 0 (n = 105)**	0 (0.0%)	7 (6.7%)	6 (5.7%)	92 (87.6%)
	**Cluster 1 (n = 139)**	0 (0.0%)	9 (6.5%)	9 (6.5%)	121 (87.1%)
	**Cluster 2 (n = 123)**	2 (1.6%)	12 (9.8%)	5 (4.1%)	104 (84.6%)
**Contralateral leg subset**	**Cluster 0 (n = 109)**	0 (0.0%)	9 (8.3%)	6 (5.5%)	94 (86.2%)
	**Cluster 1 (n = 120)**	3 (2.5%)	6 (5.0%)	6 (5.0%)	105 (87.5%)
	**Cluster 2 (n = 142)**	1 (0.7%)	15 (10.6%)	8 (5.6%)	118 (83.1%)

Values are number of players (percentage of n); The Fisher-Freeman-Halton Exact test yielded a p-value of 0.603 and 0.460 for the ipsilateral and contralateral subset, respectively; Prev/New ACL group, players with a previous ACL injury who went on to sustain a new secondary ACL injury; Prev ACL group, players with a previous ACL injury only; New ACL group, players without a previous ACL injury who went on to sustain a new primary ACL injury; No ACL group, injury free players.

Supplementary Table 15. *Cluster descriptive statistics for the Football Core 5 subset.*

		Cutting width (°)	Cutting depth (°)	Torso flexion (°)	Torso lateral flexion (°)	Torso rotation (°)
**Ipsilateral leg subset**	**Cluster 0 (n = 151)**	29.3 ± 2.9	33.8 ± 2.7	12.6 ± 8.9	15.5 ± 6.1	12.7 ± 7.5
	**Cluster 1 (n = 87)**	23.6 ± 4.0	37.7 ± 3.1	2.3 ± 9.9	20.9 ± 7.7	22.7 ± 9.9
	**Cluster 2 (n = 142)**	30.7 ± 3.2	27.5 ± 3.4	15.6 ± 9.1	6.1 ± 5.2	3.8 ± 7.7
**Contralateral leg subset**	**Cluster 0 (n = 95)**	23.4 ± 3.4	37.2 ± 3.3	2.7 ± 9.9	19.0 ± 7.3	21.2 ± 9.7
	**Cluster 1 (n = 152)**	30.5 ± 3.1	28.0 ± 3.8	15.7 ± 9.1	6.0 ± 5.0	3.8 ± 7.5
	**Cluster 2 (n = 133)**	30.1 ± 2.6	33.5 ± 3.2	12.1 ± 8.9	17.1 ± 5.8	14.6 ± 8.0

Values are means ± SD. Torso flexion: Positive values indicate torso forward flexion; Torso lateral flexion: Positive values indicate torso lateral flexion in the intended cutting direction; Torso rotation: Positive values indicate torso rotation in the intended cutting direction.

Supplementary Table 16. *The distribution of players between the three clusters in each injury group for the Football Core 5 subset.*

		Prev/New ACL group	Prev ACL group	New ACL group	No ACL group
**Ipsilateral leg subset**	**Cluster 0 (n = 151)**	1 (0.7%)	11 (7.3%)	10 (6.6%)	129 (85.4%)
	**Cluster 1 (n = 87)**	3 (3.4%)	3 (3.4%)	3 (3.4%)	78 (89.7%)
	**Cluster 2 (n = 142)**	0 (0.0%)	4 (2.8%)	10 (7.0%)	128 (90.1%)
**Contralateral leg subset**	**Cluster 0 (n = 95)**	1 (1.1%)	6 (6.3%)	4 (4.2%)	84 (88.4%)
	**Cluster 1 (n = 152)**	1 (0.7%)	2 (1.3%)	10 (6.6%)	139 (91.4%)
	**Cluster 2 (n = 133)**	1 (0.8%)	11 (8.3%)	9 (6.8%)	112 (84.2%)

Values are number of players (percentage of n); The Fisher-Freeman-Halton Exact test yielded a p-value of 0.116 and 0.094 for the ipsilateral and contralateral subset, respectively; Prev/New ACL group, players with a previous ACL injury who went on to sustain a new secondary ACL injury; Prev ACL group, players with a previous ACL injury only; New ACL group, players without a previous ACL injury who went on to sustain a new primary ACL injury; No ACL group, injury free players.

Supplementary Table 17. *Adjusted rand indices comparing the clustering results with the true labels of four different injury groupings and one sport grouping.*

		Injury groups				Sport groups
			BIN No ACL group	***BIN Prev ACL group***	***BIN New ACL group***	
**Ipsilateral leg subset**	**Core 5**	0.00	0.00	0.00	0.00	0.64
	**Handball Core 5**	0.00	0.00	0.00	0.00	n.a.
	**Football Core 5**	0.00	0.00	0.00	0.00	n.a.
**Contralateral leg subset**	**Core 5**	-0.01	-0.01	-0.01	0.00	0.63
	**Handball Core 5**	0.00	0.00	0.00	0.00	n.a.
	**Football Core 5**	0.00	0.00	0.01	0.00	n.a.

Injury groups, the four injury groups depicted in Figure 1 (main article); BIN No ACL group, No ACL group vs. all other groups; BIN Prev ACL group, Prev/New ACL group and Prev ACL group vs. all other groups; BIN New ACL group, Prev/New ACL group and New ACL group vs. all other groups; Sport groups, Handball vs. football players.

3. Results for the 4-cluster models

Supplementary Table 18. *Cluster descriptive statistics for the Core 5 subset.*

		Cutting width (°)	Cutting depth (°)	Torso flexion (°)	Torso lateral flexion (°)	Torso rotation (°)
**Ipsilateral leg subset**	**Cluster 0 (n = 188)**	30.6 ± 3.3	28.5 ± 3.6	16.0 ± 9.0	8.0 ± 6.1	5.7 ± 8.2
	**Cluster 1 (n = 189)**	26.7 ± 4.1	36.2 ± 2.9	8.0 ± 8.8	18.5 ± 7.1	17.6 ± 9.4
	**Cluster 2 (n = 178)**	21.3 ± 3.3	24.3 ± 4.6	-5.2 ± 8.0	-3.9 ± 6.2	-5.6 ± 10.2
	**Cluster 3 (n = 192)**	19.6 ± 4.1	27.4 ± 4.2	-13.5 ± 8.1	6.8 ± 6.2	13.1 ± 10.1
**Contralateral leg subset**	**Cluster 0 (n = 158)**	25.9 ± 4.2	36.6 ± 3.0	6.2 ± 9.0	19.3 ± 6.6	20.1 ± 9.1
	**Cluster 1 (n = 191)**	21.3 ± 3.3	24.8 ± 4.5	-4.6 ± 7.7	-3.9 ± 5.9	-5.3 ± 10.6
	**Cluster 2 (n = 187)**	19.8 ± 4.1	26.9 ± 4.3	-13.9 ± 8.2	6.7 ± 5.8	13.1 ± 9.5
	**Cluster 3 (n = 215)**	30.7 ± 3.0	29.3 ± 4.0	15.7 ± 9.0	9.2 ± 6.3	6.7 ± 8.0

Values are means ± SD. Torso flexion: Positive values indicate torso forward flexion; Torso lateral flexion: Positive values indicate torso lateral flexion in the intended cutting direction; Torso rotation: Positive values indicate torso rotation in the intended cutting direction.

Supplementary Table 19. *The distribution of players between the four clusters in each injury group for the Core 5 subset.*

		Prev/New ACL group	Prev ACL group	New ACL group	No ACL group
**Ipsilateral leg subset**	**Cluster 0 (n = 188)**	0 (0.0%)	8 (4.3%)	13 (6.9%)	167 (88.8%)
	**Cluster 1 (n = 189)**	5 (2.6%)	12 (6.3%)	9 (4.8%)	163 (86.2%)
	**Cluster 2 (n = 178)**	0 (0.0%)	11 (6.2%)	13 (7.3%)	154 (86.5%)
	**Cluster 3 (n = 192)**	1 (0.5%)	15 (7.8%)	8 (4.2%)	168 (87.5%)
**Contralateral leg subset**	**Cluster 0 (n = 158)**	2 (1.3%)	10 (6.3%)	8 (5.1%)	138 (87.3%)
	**Cluster 1 (n = 191)**	1 (0.5%)	17 (8.9%)	14 (7.3%)	159 (83.2%)
	**Cluster 2 (n = 187)**	3 (1.6%)	12 (6.4%)	7 (3.7%)	165 (88.2%)
	**Cluster 3 (n = 215)**	1 (0.5%)	10 (4.7%)	14 (6.5%)	190 (88.4%)

Values are number of players (percentage of n); The Fisher-Freeman-Halton Exact test yielded a p-value of 0.173 and 0.563 for the ipsilateral and contralateral subset, respectively; Prev/New ACL group, players with a previous ACL injury who went on to sustain a new secondary ACL injury; Prev ACL group, players with a previous ACL injury only; New ACL group, players without a previous ACL injury who went on to sustain a new primary ACL injury; No ACL group, injury free players.

Supplementary Table 20. *The distribution of players between the four clusters in each sport group for the Core 5 subset.*

		**Football**	**Handball**
**Ipsilateral leg subset**	**Cluster 0 (n = 188)**	182 (96.8%)	6 (3.2%)
	**Cluster 1 (n = 189)**	182 (96.3%)	7 (3.7%)
	**Cluster 2 (n = 178)**	5 (2.8%)	173 (97.2%)
	**Cluster 3 (n = 192)**	11 (5.7%)	181 (94.3%)
**Contralateral leg subset**	**Cluster 0 (n = 158)**	152 (96.2%)	6 (3.8%)
	**Cluster 1 (n = 191)**	8 (4.2%)	183 (95.8%)
	**Cluster 2 (n = 187)**	9 (4.8%)	178 (95.2%)
	**Cluster 3 (n = 215)**	211 (98.1%)	4 (1.9%)

Values are number of players (percentage of n); The Fisher-Freeman-Halton Exact test yielded a p-value of < 0.001 and < 0.001 for the ipsilateral and contralateral subset, respectively.

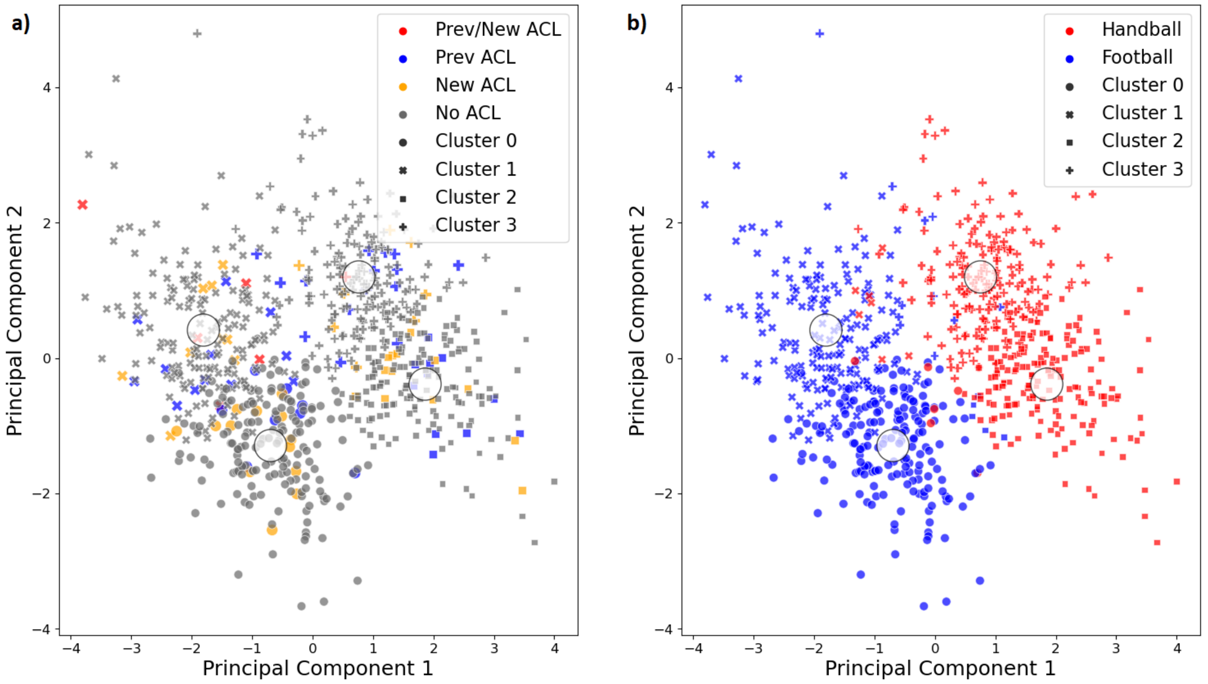

Supplementary Figure 4*. Visualization of the four clusters (Cluster 0, Cluster 1, Cluster 2 and Cluster 3) as well as the true labels of* ***(A)*** *the injury groups and* ***(B)*** *the sport groups for the Core 5 subset (ipsilateral leg subset). The first two principal components of the principal component analysis, which explain the highest amount of variance in the original data, are plotted against each other. Prev/New ACL group, players with a previous ACL injury who went on to sustain a new secondary ACL injury; Prev ACL group, players with a previous ACL injury only; New ACL group, players without a previous ACL injury who went on to sustain a new primary ACL injury; No ACL group, injury free players.*

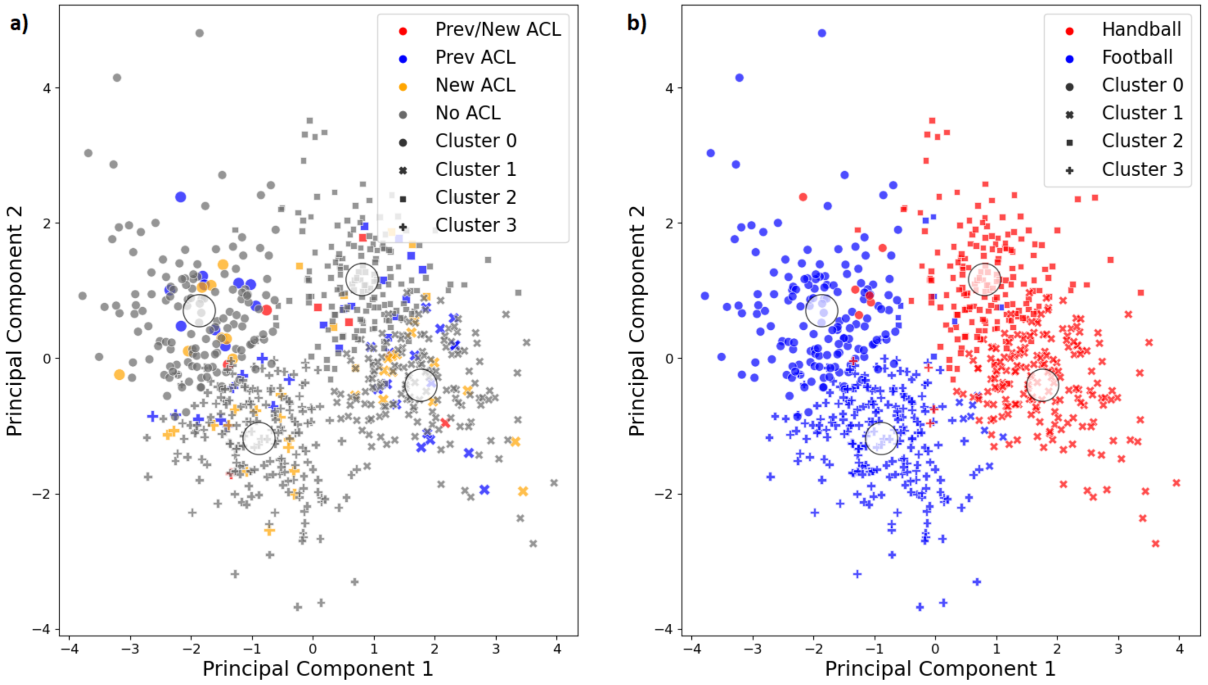

Supplementary Figure 5*. Visualization of the four clusters (Cluster 0, Cluster 1, Cluster 2 and Cluster 3) as well as the true labels of* ***(A)*** *the injury groups and* ***(B)*** *the sport groups for the Core 5 subset (contralateral leg subset). The first two principal components of the principal component analysis, which explain the highest amount of variance in the original data, are plotted against each other. Prev/New ACL group, players with a previous ACL injury who went on to sustain a new secondary ACL injury; Prev ACL group, players with a previous ACL injury only; New ACL group, players without a previous ACL injury who went on to sustain a new primary ACL injury; No ACL group, injury free players.*

Supplementary Table 21. *Cluster descriptive statistics for the Handball Core 5 subset.*

		Cutting width (°)	Cutting depth (°)	Torso flexion (°)	Torso lateral flexion (°)	Torso rotation (°)
**Ipsilateral leg subset**	**Cluster 0 (n = 98)**	17.2 ± 3.3	30.0 ± 3.1	-12.1 ± 8.3	5.2 ± 5.6	13.6 ± 11.1
	**Cluster 1 (n = 112)**	21.3 ± 3.0	26.9 ± 3.5	-0.8 ± 6.8	-4.2 ± 6.2	-2.6 ± 10.1
	**Cluster 2 (n = 90)**	23.0 ± 3.0	24.3 ± 3.2	-13.9 ± 7.9	9.1 ± 5.4	11.2 ± 8.9
	**Cluster 3 (n = 67)**	20.3 ± 3.3	20.5 ± 3.5	-11.7 ± 7.7	-4.0 ± 5.5	-8.4 ± 11.1
**Contralateral leg subset**	**Cluster 0 (n = 90)**	17.6 ± 3.5	29.4 ± 3.3	-14.6 ± 6.4	6.7 ± 6.2	15.6 ± 11.3
	**Cluster 1 (n = 104)**	21.3 ± 3.5	28.4 ± 3.6	-1.2 ± 6.1	-0.0 ± 6.4	3.5 ± 9.8
	**Cluster 2 (n = 95)**	22.2 ± 3.4	22.5 ± 3.7	-16.6 ± 7.2	5.5 ± 5.7	6.2 ± 9.6
	**Cluster 3 (n = 82)**	20.4 ± 3.1	22.8 ± 3.7	-4.5 ± 6.5	-7.3 ± 5.4	-10.4 ± 11.8

Values are means ± SD. Torso flexion: Positive values indicate torso forward flexion; Torso lateral flexion: Positive values indicate torso lateral flexion in the intended cutting direction; Torso rotation: Positive values indicate torso rotation in the intended cutting direction.

Supplementary Table 22. *The distribution of players between the four clusters in each injury group for the Handball Core 5 subset.*

		Prev/New ACL group	Prev ACL group	New ACL group	No ACL group
**Ipsilateral leg subset**	**Cluster 0 (n = 98)**	1 (1.0%)	7 (7.1%)	5 (5.1%)	85 (86.7%)
	**Cluster 1 (n = 112)**	1 (0.9%)	5 (4.5%)	8 (7.1%)	98 (87.5%)
	**Cluster 2 (n = 90)**	0 (0.0%)	8 (8.9%)	4 (4.4%)	78 (86.7%)
	**Cluster 3 (n = 67)**	0 (0.0%)	8 (11.9%)	3 (4.5%)	56 (83.6%)
**Contralateral leg subset**	**Cluster 0 (n = 90)**	2 (2.2%)	5 (5.6%)	4 (4.4%)	79 (87.8%)
	**Cluster 1 (n = 104)**	1 (1.0%)	9 (8.7%)	7 (6.7%)	87 (83.7%)
	**Cluster 2 (n = 95)**	0 (0.0%)	7 (7.4%)	5 (5.3%)	83 (87.4%)
	**Cluster 3 (n = 82)**	1 (1.2%)	9 (11.0%)	4 (4.9%)	68 (82.9%)

Values are number of players (percentage of n); The Fisher-Freeman-Halton Exact test yielded a p-value of 0.752 and 0.897 for the ipsilateral and contralateral subset, respectively; Prev/New ACL group, players with a previous ACL injury who went on to sustain a new secondary ACL injury; Prev ACL group, players with a previous ACL injury only; New ACL group, players without a previous ACL injury who went on to sustain a new primary ACL injury; No ACL group, injury free players.

Supplementary Table 23. *Cluster descriptive statistics for the Football Core 5 subset.*

		Cutting width (°)	Cutting depth (°)	Torso flexion (°)	Torso lateral flexion (°)	Torso rotation (°)
**Ipsilateral leg subset**	**Cluster 0 (n = 91)**	32.4 ± 2.2	26.2 ± 3.3	18.9 ± 8.6	6.9 ± 5.2	4.6 ± 8.3
	**Cluster 1 (n = 79)**	23.2 ± 3.6	37.8 ± 3.1	2.0 ± 9.7	20.5 ± 7.2	23.0 ± 9.7
	**Cluster 2 (n = 96)**	27.4 ± 2.8	31.4 ± 2.9	8.8 ± 8.3	6.5 ± 4.9	5.6 ± 7.5
	**Cluster 3 (n = 114)**	30.0 ± 2.6	34.2 ± 2.9	13.9 ± 8.4	18.8 ± 5.5	14.6 ± 7.8
**Contralateral leg subset**	**Cluster 0 (n = 85)**	23.4 ± 3.5	37.6 ± 3.2	2.3 ± 9.8	20.2 ± 6.6	22.5 ± 9.2
	**Cluster 1 (n = 91)**	32.4 ± 2.2	26.2 ± 3.3	19.0 ± 8.6	6.9 ± 5.2	4.4 ± 8.0
	**Cluster 2 (n = 110)**	30.5 ± 2.4	33.8 ± 3.0	13.0 ± 8.5	18.3 ± 5.5	15.1 ± 8.2
	**Cluster 3 (n = 94)**	27.3 ± 2.7	31.4 ± 2.9	9.5 ± 8.5	6.7 ± 5.0	5.9 ± 7.6

Values are means ± SD. Torso flexion: Positive values indicate torso forward flexion; Torso lateral flexion: Positive values indicate torso lateral flexion in the intended cutting direction; Torso rotation: Positive values indicate torso rotation in the intended cutting direction.

Supplementary Table 24. *The distribution of players between the four clusters in each injury group for the Football Core 5 subset.*

		Prev/New ACL group	Prev ACL group	New ACL group	No ACL group
**Ipsilateral leg subset**	**Cluster 0 (n = 91)**	0 (0.0%)	1 (1.1%)	7 (7.7%)	83 (91.2%)
	**Cluster 1 (n = 79)**	3 (3.8%)	3 (3.8%)	3 (3.8%)	70 (88.6%)
	**Cluster 2 (n = 96)**	0 (0.0%)	6 (6.3%)	4 (4.2%)	86 (89.6%)
	**Cluster 3 (n = 114)**	1 (0.9%)	8 (7.0%)	9 (7.9%)	96 (84.2%)
**Contralateral leg subset**	**Cluster 0 (n = 85)**	1 (1.2%)	6 (7.1%)	4 (4.7%)	74 (87.1%)
	**Cluster 1 (n = 91)**	1 (1.1%)	0 (0.0%)	7 (7.7%)	83 (91.2%)
	**Cluster 2 (n = 110)**	1 (0.9%)	10 (9.1%)	8 (7.3%)	91 (82.7%)
	**Cluster 3 (n = 94)**	0 (0.0%)	3 (3.2%)	4 (4.3%)	87 (92.6%)

Values are number of players (percentage of n); The Fisher-Freeman-Halton Exact test yielded a p-value of 0.145 and 0.062 for the ipsilateral and contralateral subset, respectively; Prev/New ACL group, players with a previous ACL injury who went on to sustain a new secondary ACL injury; Prev ACL group, players with a previous ACL injury only; New ACL group, players without a previous ACL injury who went on to sustain a new primary ACL injury; No ACL group, injury free players.

Supplementary Table 25. *Adjusted rand indices comparing the clustering results with the true labels of four different injury groupings and one sport grouping.*

		Injury groups	*BIN No ACL group*	*BIN Prev ACL group*	*BIN New ACL group*	Sport groups
**Ipsilateral leg subset**	**Core 5**	0.00	0.00	0.00	0.00	0.42
	**Handball Core 5**	0.00	0.00	0.00	0.00	n.a.
	**Football Core 5**	0.00	0.00	0.00	0.00	n.a.
**Contralateral leg subset**	**Core 5**	0.00	0.00	0.00	0.00	0.44
	**Handball Core 5**	0.00	0.00	0.00	0.00	n.a.
	**Football Core 5**	0.00	0.00	0.00	0.00	n.a.

Injury groups, the four injury groups depicted in Figure 1 (main article); BIN No ACL group, No ACL group vs. all other groups; BIN Prev ACL group, Prev/New ACL group and Prev ACL group vs. all other groups; BIN New ACL group, Prev/New ACL group and New ACL group vs. all other groups; Sport groups, Handball vs. football players.
